# Functional switching of NPR1 between chloroplast and nucleus for adaptive response to salt stress

**DOI:** 10.1038/s41598-020-61379-3

**Published:** 2020-03-09

**Authors:** So Yeon Seo, Soo Jin Wi, Ky Young Park

**Affiliations:** Department of Biology, Sunchon National University, Sunchon, Chonnam Republic of Korea

**Keywords:** Plant signalling, Salt

## Abstract

Salt stress causes rapid accumulation of nonexpressor of pathogenesis-related genes 1 (NPR1) protein, known as the redox-sensitive transcription coactivator, which in turn elicits many adaptive responses. The NPR1 protein transiently accumulates in chloroplast stroma under salt stress, which attenuates stress-triggered down-regulation of photosynthetic capability. We observed that oligomeric NPR1 in chloroplasts and cytoplasm had chaperone activity, whereas monomeric NPR1 in the nucleus did not. Additionally, NPR1 overexpression resulted in reinforcement of morning-phased and evening-phased circadian clock. NPR1 overexpression also enhanced antioxidant activity and reduced stress-induced reactive oxygen species (ROS) generation at early stage, followed with transcription levels for ROS detoxification. These results suggest a functional switch from a molecular chaperone to a transcriptional coactivator, which is dependent on subcellular localization. Our findings imply that dual localization of NPR1 is related to proteostasis and redox homeostasis in chloroplasts for emergency restoration as well as transcriptional coactivator in the nucleus for adaptation to stress.

## Introduction

Chloroplasts are particularly vulnerable to environmental disturbances, because of oxygenic photosynthesis^1^, after which the generation of reactive oxygen species (ROS)^2^ might occur as a more serious phenomenon^3^. Even though ROS play an important role as signaling molecules and inducers in the adaption of plants to abiotic stress, they are also toxic byproducts of stress metabolism^4^. Chloroplasts act as sensors of the present environmental situation^5^ and produce diverse signals communicating the functionality of the photosynthetic apparatus to the nucleus, which is defined as retrograde signaling^6^.

Recent genomic technologies provide growing evidence that ROS generation is one of the most common responses to different stresses in plants, representing various signaling pathways come together^7,8^. Because the rapid generation of ROS represents a common plant response to almost all environmental challenges^4,9^, it is suggested that ROS and the redox system in chloroplasts represent primary sources within the plant signaling battery. This hypothesis implies there are interactions between ROS and other signaling components^4^ such as redox homeostasis, plant hormones, and transcription factors^4^.

Sunlight for photosynthesis is available only for a limited period within the 24 h day. The rhythmic and predictable alteration of solar energy has driven the evolution of the circadian clock, which is integrated with signals within chloroplasts^4,10^. Nuclear-encoded transcripts for chloroplast proteins may be related to the circadian regulation of chloroplasts^4,11^.

Proper protein folding and localization are critical for cellular protein function. However, cells are exposed to environmental stresses, which makes them susceptive to nonnative condition that ultimately can result in misfolding and aggregation^4,12^. In addition, ROS or oxidized small molecules are involved in aberrant modifications of protein structure, which eventually leads to toxic effects on cells^13^. Therefore, chloroplasts have well developed mechanisms protecting against protein modifications via molecular chaperones that bind reversibly to unfolded and misfolded proteins, thereby maintaining native protein conformation. Therefore, chaperones are now considered as powerful buffers for multiple stress resistance^14^.

Numerous studies have revealed that nonexpressor of pathogenesis-related genes 1 (NPR1) protein is a master regulator of plant immunity with salicylic acid (SA)-mediated defense responses and systemic acquired resistance (SAR) in Arabidopsis^15–17^. In unstressed cells, NPR1 is mostly localized in the cytoplasm as a tetrameric complex with redox-sensitive intermolecular disulfide bonds^15^. Pathogen infection triggers alteration of cellular reduction potential, thereby reducing NPR1 tetramer into a monomer via breakage of disulfide bonds, after which NPR1 monomer is imported into the nucleus to function as a coactivator of gene transcription^18^. Nuclear NPR1 interacts with transcription factor (TF) members of the TGA class of basic domain/leucine zipper^19^. However, there are exceptional cases in which nuclear localization of NPR1 is not a requirement for SA-dependent gene regulation^20^, indicating SA-activated cytosolic NPR1 has a novel function beyond transcriptional coactivator in nucleus^18^.

Here, we observed that NPR1, a so-called nucleocytoplasmic protein, transiently accumulated in the chloroplast stroma of *Nicotiana tabacum* and *Arabidopsis thaliana* under salt stress and SA treatment. NPR1 in the chloroplasts was shown to have the function of chaperone activity and redox regulation as well as transcriptional coactivator in the nucleus in response to salt stress, suggesting NPR1 might be involved in proteostasis and redox homeostasis. The novel features of chloroplasts related with NPR1 are very effective in promoting adaptation against abiotic/biotic stress and developmental regulation in plants.

## Results

### Enhanced expression of tobacco NPR1 induces tolerance to salt stress

**Figure 1:**
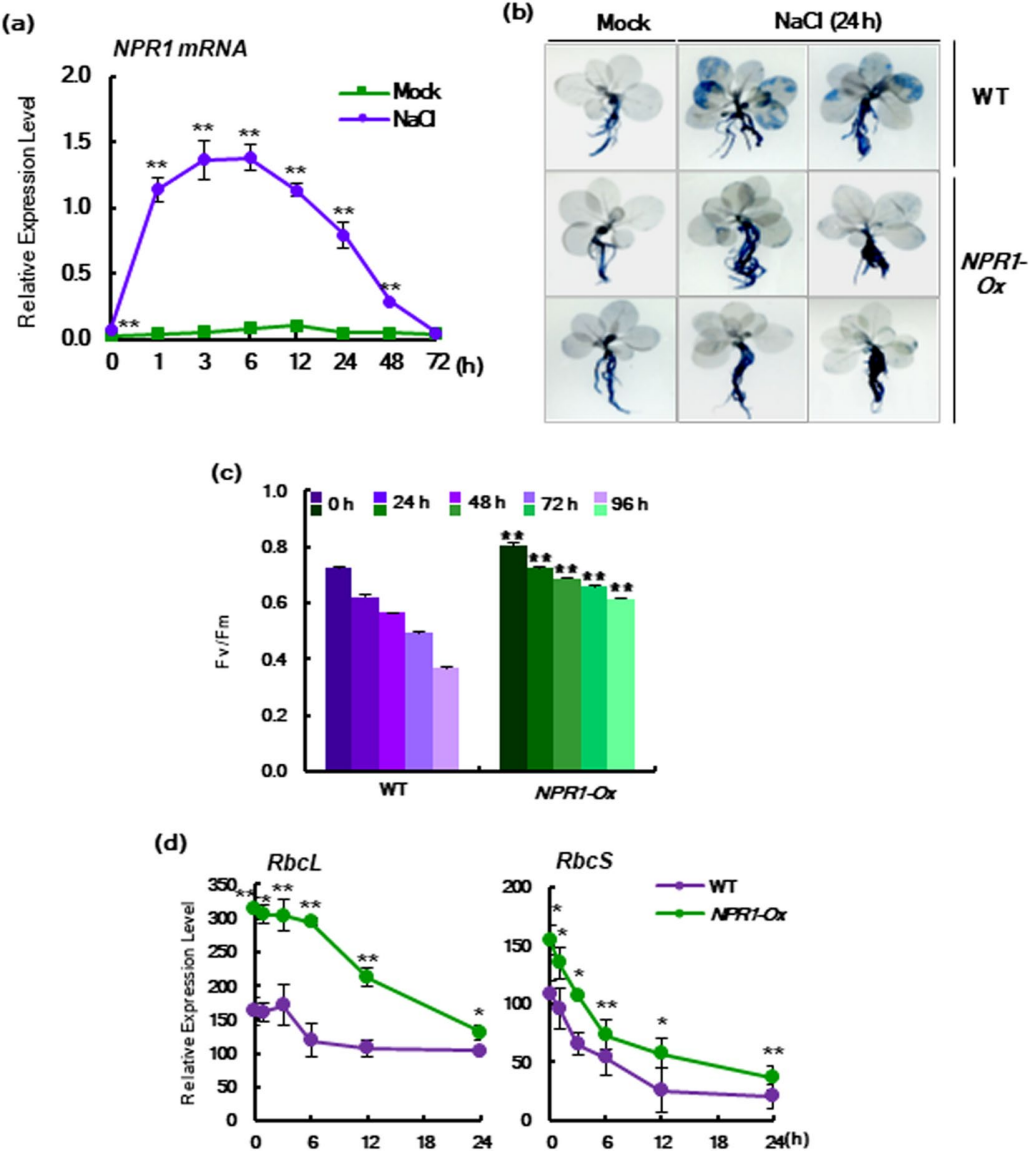
Expression of *NPR1* mRNA is transiently increased while overexpression of NPR1 has positive effects on stress tolerance. (**a**) Expression of *NPR1* mRNA was detected in WT leaves under high salinity conditions (200 mM NaCl). (**b**) Necrotic areas in salt-stressed whole plants were stained with trypan blue in WT and *NPR1-Ox* plants. (**c**) The maximal photochemical efficiency of photosystem II (PC II) (*Fv*/*Fm*) was measured after salt stress. *Fv*/*Fm* values were expressed as means ± SD with n = 10. (**d**) Relative expression ratios of *RbcL* and *RbcS* in *NPR1-Ox* versus WT under high salinity conditions. Transcription levels were measured by real-time qRT-PCR. Data represent from five (**a**) or three (**b,d**) independent experiments with n = 3.

Although it is well known that NPR1 protein is a key transcriptional coactivator in broad-spectrum immunity of plants against phytopathogens^21^, NPR1 has not been fully characterized in relation to abiotic stresses. Here, we observed that the transcription levels of NPR1 rapidly increased from 1 h to 6 h, after which they gradually returned to basal levels in leaves of *Nicotiana tabacum* after salt stress (Fig. 1a). To investigate the physiological significances of NPR1 in response to salt stress, we established tobacco transgenic plants with *p*35*S*-driven *NPR1* overexpression (*NPR1-Ox*)^22^. *NPR1-Ox* plants showed greater tolerance to salt stress, as determined by trypan blue staining for cell death^22^ (Fig. 1b) as well as maximal photochemical efficiency of photosystem (PS) II (*Fv/Fm*)^23^ based on chlorophyll fluorescence using a PAM 2000 Photosynthesis Yield Analyzer (Walz, Germany) (Fig. 1c). Under salt stress, *Fv/Fm* was reduced by 49% in WT tobacco plants after 96 h compared with the unstressed control (0 h). However, the ratio of *Fv/Fm* was reduced only by 24% after 96 h of salt stress in *NPR1-Ox* leaves.

Quantitative real-time RT-PCR (qRT-PCR) was performed with ribulose-1,5-bisphosphate carboxylase (RubisCO) genes responsible for photosynthesis^24^. Stress-triggered transcriptional down-regulation of chloroplast-encoded large subunits (*RbcL*) and nucleus-encoded small subunits (*RbcS*) was mitigated in *NPR1-Ox* compared to WT (Fig. 1d). *RbcL* transcription levels were maintained until 6 h under stress conditions in *NPR1-Ox* but until 3 h in WT. Specifically, the *RbcL* transcription level was higher in *NPR1-Ox* than in WT upon salt stress. These results indicate NPR1 plays a specific role against rapid down-regulation of chloroplast-encoded gene expression in the early stage in salt stress. Nucleus-encoded *RbcS* transcription was down-regulated gradually in *NPR1-Ox* and WT tobacco leaves under salt stress, and lower levels were observed in WT than in *NPR1-Ox*.

**Figure 2:**
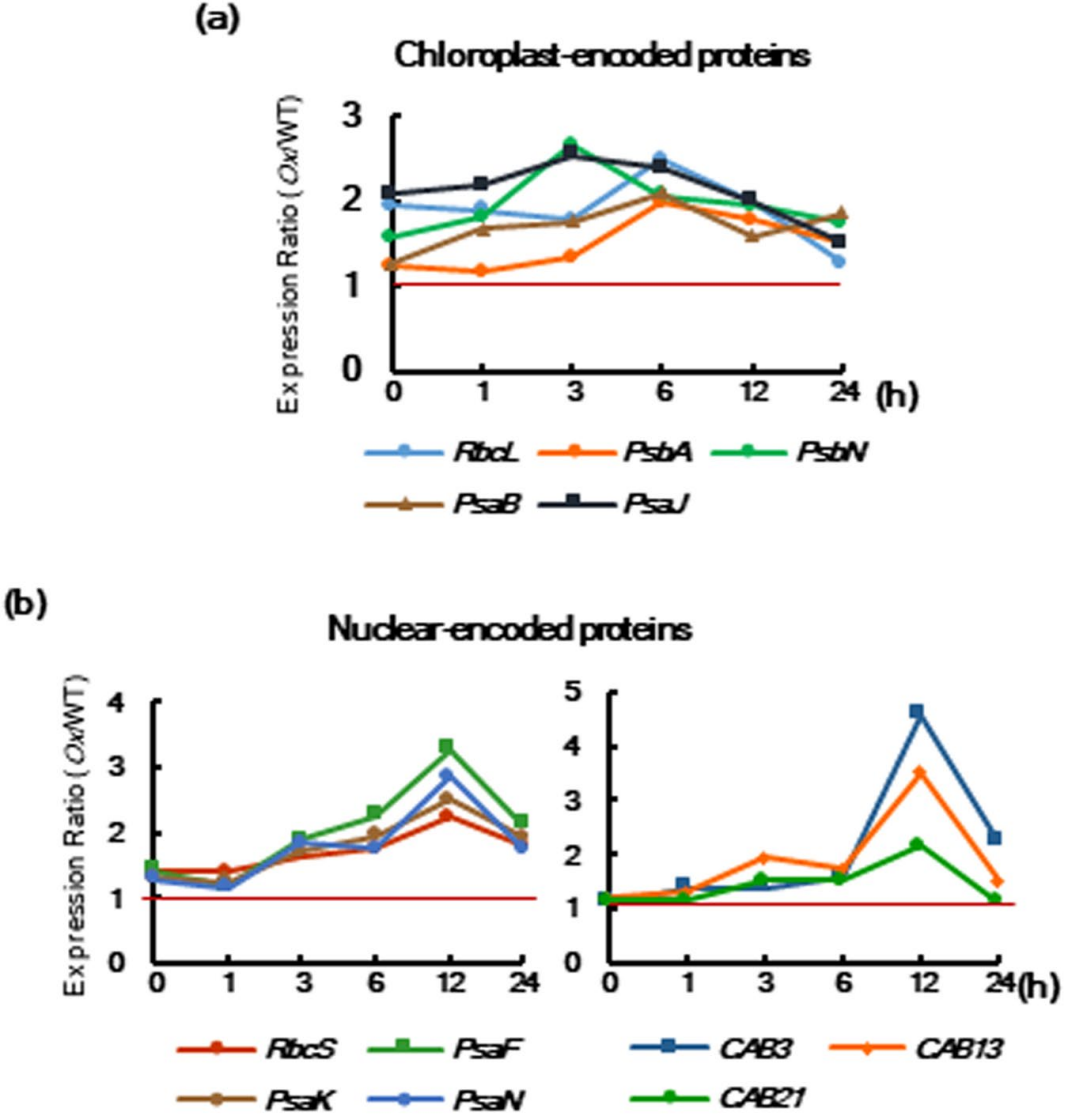
NPR1 transiently increases expression of genes related to the photosynthetic apparatus and enzymes after salt stress. Relative expression ratios of transcription levels of chloroplast-encoded (**a**) and nuclear-encoded (**b**) proteins for the photosynthetic apparatus and enzymes in *NPR1-Ox* versus WT after salt stress. Relative expression level of each gene was determined by real-time qRT-PCR with normalization of the reference gene β-actin. The expression ratio value was computed based on the relative expression level of each gene in *NPR1-Ox* versus WT after salt stress. Red line is indicated the ratio value 1. Data represent from three independent experiments with n = 3.

To elucidate the physiological functions of chloroplast and nuclear NPR1 in response to salt stress, we compared the transcription patterns of chloroplast- and nuclear-encoded genes for photosynthesis-related proteins between WT and *NPR1-Ox* upon salt stress. Real-time qRT-PCR was performed using genes for RubisCO and core complex and antenna proteins of PS I and II. The transcriptional ratios of *NPR1-Ox* to WT were obtained on the basis of each relative expression value for chloroplast- and nucleus-encoded genes using β-actin as the reference gene. Transcriptional ratios of chloroplast- and nucleus-encoded genes above 1 indicated higher gene expression in *NPR1-Ox* compared with WT (Fig. 2a,b). Almost all chloroplast-encoded transcripts ratios peaked from 3 to 6 h after salt stress, whereas transcript ratios of nuclear-encoded *RbcS* and *PsaF* significantly increased from 6 to 12 h.

Salt stress slightly increased the transcription level of *PsbA* at 3 h in WT and *NPR1-Ox* plants, after which *PsbA* transcription was significantly reduced (Supplementary Fig. 1). On the other hand, the transcription levels of *PsbN*, *PsaB*, and *PsaJ* were immediately reduced after salt stress in both WT and *NPR1-Ox* plants. Overall, higher levels of all transcripts were maintained during the entire period in *NPR1-Ox* compared to WT. All tested nuclear-encoded genes, including *PsaK*, *PsaN*, *CAB3*, *CAB13*, and *CAB21*, showed a peak of transcript ratios for *NPR1-Ox* to WT at 12 h (Fig. 2b, Supplementary Fig. 2).

The production of sugars by photosynthesis is the main metabolic outcome of circadian clock in plants^17,25^. It has recently been reported that the redox rhythm is closely associated with the circadian clock^26^, and NPR1 regulates transcription of the circadian clock genes without changing the period^27^. The core circadian oscillator is defined, in which the sequential expression of transcription factor genes is regulated by a negative feedback loop with daily time-keeping across the 24-h cycle^28^. The core loop consists of interconnected transcription factors, which include the morning clock genes of LATE ELONGATED HYPOCOTYL (LHY) and CIRCADIAN CLOCK ASSOCIATED (CCA1) and the evening clock gene of TIMING OF CAB2 EXPRESSION 1 (TOC1)^26,29^.

**Figure 3:**
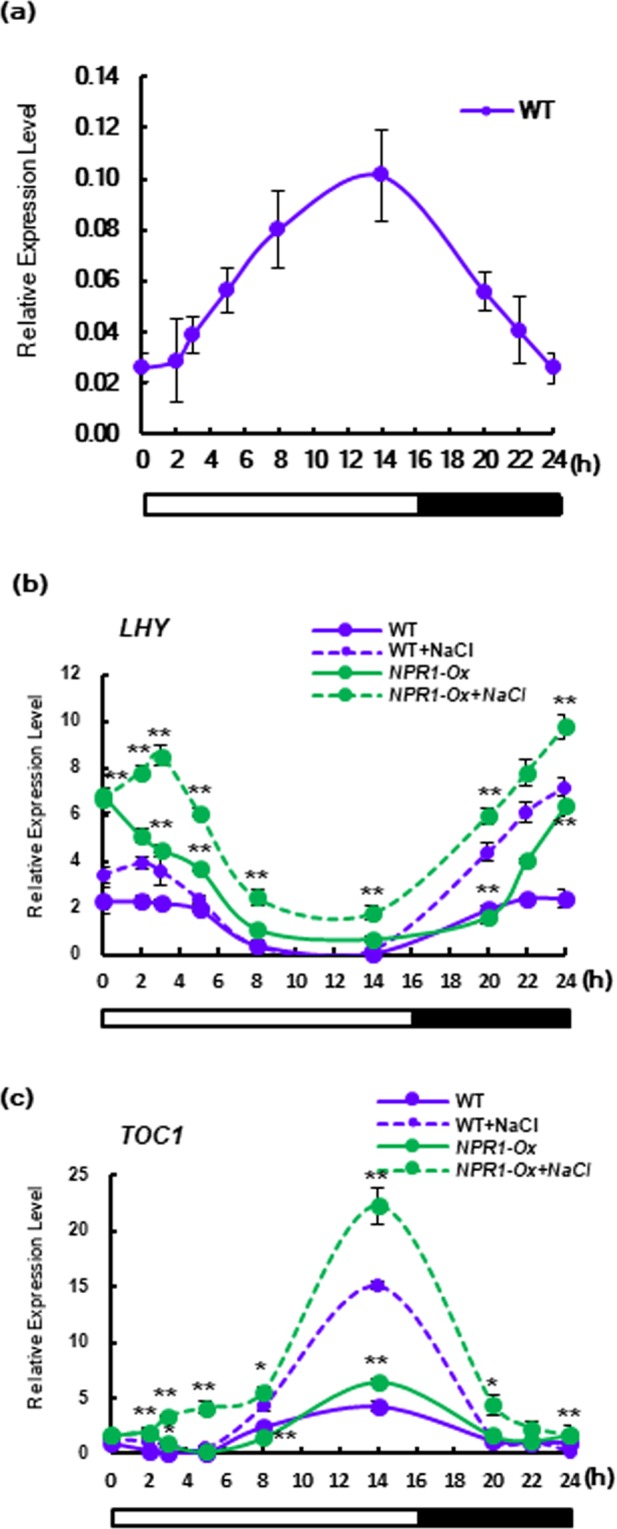
Kinetics of circadian rhythms-related gene transcription in response to salt stress. Profile of transcription level of *NPR1*, *LHY* and *TOC1* was measured by real-time qPCR in response to salt stress induced by 200 mM NaCl in leaves of WT and *NPR1-Ox* transgenic plants. Transcription levels were expressed relative to the reference gene β-actin after qPCR. Relative mRNA expression levels were expressed as means ± SD. An asterisk indicates significant difference between WT and transgenic plants with stress-treated or untreated cases (one asterisk (P < 0.05) or two asterisks (P < 0.01)). Data represent from four independent experiments with n = 3.

To elucidate the physiological relationship between circadian clock and NPR1 in response to salt stress, qPCR was performed with genes for circadian rhythm. We found a circadian pattern of NPR1 transcription with a peak before evening in WT tobacco plants under 16 L/8D cycles without any treatment, suggesting the pattern was linked to the clock-regulated redox rhythm (Fig. 3a). Higher transcription of *LHY* and *TOC1* was detected in *NPR1-Ox* than WT, and the transcription levels of both genes were significantly increased in salt stressed-tobacco plants relative to mock treated-tobacco plants (Fig. 3b,c). Our results indicated that NPR1 induced substantial increases in amplitude and transcript expression of *LHY* and *TOC1* without changing circadian period and phase under salt stress. Taken together, these results suggest that NPR1 results in reinforcement of morning-phased and evening-phased clock, implying that NPR1 functions as a redox-sensitive amplifier by conveying stress-induced redox information to the chloroplasts.

### NPR1 is targeted to chloroplasts under salt stress in *Nicotiana tabacum* and *Arabidopsis thaliana*

**Figure 4:**
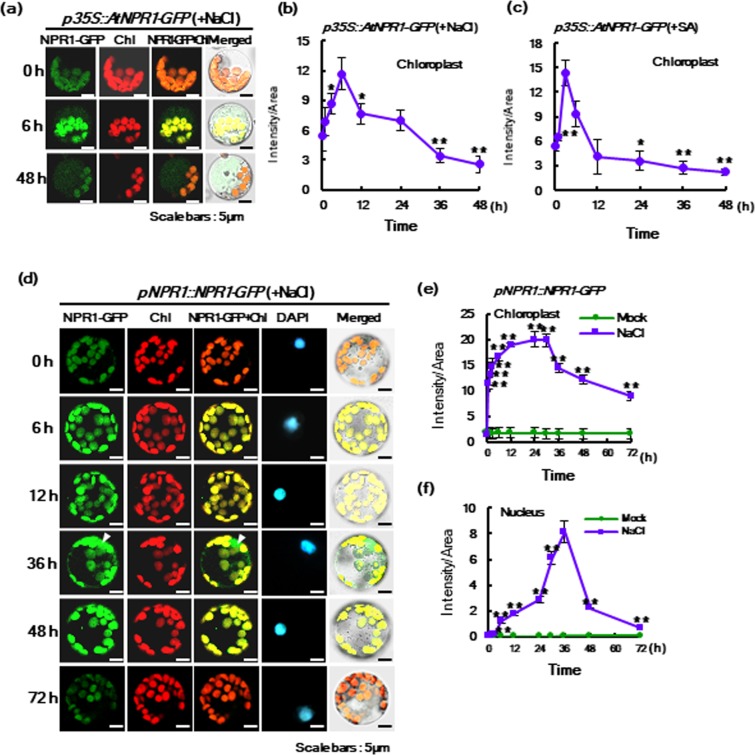
NPR1 protein is dually localized to chloroplasts and the nucleus in *Arabidopsis taliana* and *Nicotiana tabacum* after salt stress. (**a**) Confocal laser scanning microscopy (CLSM) images of GFP fluorescence were photographed for *p35S::AtNPR1-GFP* transient expression into mesophyll protoplasts under high salinity conditions of 100 mM NaCl. (**b**,**c**) Fluorescence intensity of AtNPR1-GFP in chloroplasts after transient expression of *p35S::AtNPR1-GFP* into mesophyll protoplasts of WT Arabidopsis leaves under salt stress (**b**) and SA treatment (**c**). Quantitation of fluorescence intensity of AtNPR1-GFP was calculated using ImageJ software. (**d)** CLSM images of GFP fluorescence were photographed for *pNPR1::NPR1-GFP* stable transgenic tobacco leaves under salt stress with 200 mM NaCl. (**e**,**f)** Fluorescence intensity of NPR1-GFP in chloroplasts (**e**) and the nucleus (**f**) of protoplasts from *pNPR1::NPR1-GFP* transgenic plants under high salinity conditions. Data represent from four (**a–c**) or five (**d–f**) independent experiments with n = 20.

Because NPR1 had prominent effects on the transcription of chloroplast-encoded proteins for photosynthesis under salt stress (Fig. 2a), we preferentially determined the localization of NPR1 protein using the fusion protein construct of *p*35*S*-driven Arabidopsis *NPR1* combined with green fluorescence protein (*GFP*) (*p*35*S::AtNPR1-GFP*). It is well known that NPR1 is a nucleocytoplasmic protein in Arabidopsis^15^. However, after transient expression in mesophyll protoplasts from Arabidopsis leaves, AtNPR1-GFP was astonishingly detected in chloroplasts under salt stress (Fig. 4a,b, Supplementary Fig. 3a) and SA treatment (Fig. 4c, Supplementary Fig. 3b), which reached a peak at 6 h for salt stress and at 3 h for SA treatment.

After peak of *AtNPR1-GFP* in Arabidopsis chloroplasts under high salt treatment, NPR1-GFP disappeared gradually in chloroplasts (Fig. 4a,b, Supplementary Fig. 3a). In the case of SA treatment, which many researchers have studied mainly after 24 h or 48 h, we observed that NPR1-GFP was more rapidly imported into chloroplasts and then disappeared more rapidly back down to the initial level at 12 h (Fig. 4c, Supplementary Fig. 3b). The result of chloroplast localization of NPR1 was very surprising and has not been reported previously. Most NPR1 studies have examined the intracellular localization of NPR1 after 24 h of pathogen infection or SA treatment, indicating that import of NPR1 into chloroplasts at an early time point after abiotic and biotic stresses has not been investigated.

To better investigate the localization and functions of NPR1 under abiotic stress, we established stable transgenic tobacco plants expressing GFP fusion construct with tobacco NPR1 driven by a 0.8-kb region of the NPR1 promoter (*pNPR1::NPR1-GFP*)^22^. NPR1-GFP accumulated in chloroplasts of mesophyll protoplasts from this transgenic tobacco plant (Fig. 4d).

The fluorescence intensity of GFP was quantified by ImageJ over the defined areas of the chloroplasts with red autofluorescence, the nucleus with DAPI staining and the cytoplasm excluding the nucleus and the chloroplasts. In response to salt stress, NPR1-GFP rapidly accumulated in chloroplasts from 1 h and then reached a peak from 24 h to 30 h, after which it gradually decreased (Fig. 4e). However, this protein appeared relatively late in the nucleus from 12 h and peaked to a significant level at 36 h, after which it rapidly disappeared (Fig. 4f). Some fluorescence was measured in chloroplasts even without stress, implying that some level of NPR1 was already present under unstressed control conditions.

Fluorescence of GFP alone was detected in the cytoplasm, nucleus, cell membrane, and cell wall in 35 *S::GFP* plants and was not merged with red autofluorescence from chloroplasts in mesophyll cells and guard cells, indicating GFP alone did not enter chloroplasts rarely in tobacco leaves (Supplementary Fig. 4a,b). On the other hand, the level of NPR1-GFP was enhanced more quickly and significantly in chloroplasts of mesophyll protoplasts and guard cells of 35 *S CaMV* promoter-driven and native *NPR1* promoter-driven *NPR1-GFP* transgenic tobacco plants (Fig. 4d–f, Supplementary Fig. 4c,d). Even though NPR1-GFP was significantly localized to the inner wall and chloroplasts of guard cells in *p*35*S::NPR1-GFP* plants under unstressed conditions, it was quickly targeted to chloroplasts after salt stress (Supplementary Fig. 4d). Merged images of NPR1-GFP fluorescence and red autofluorescence of chloroplasts show a strong yellow color, indicating that a large amount of NPR1-GFP was accumulated in the chloroplasts of guard cells after 6 h of salt stress, after which it gradually decreased. Although native *NPR1* promoter-driven NPR1-GFP showed a peak in the nucleus at 30–36 h after salt stress (Fig. 4f), p35*S*-driven NPR1-GFP reached a high level more rapidly before 12 h in salt-stressed mesophyll protoplasts and guard cells (Supplementary Fig. 4c,d). This time gap can be attributed to the fact that NPR1 protein already exists in *p35S::NPR1-GFP* plants. Taken together, we first discovered that NPR1 translocated to chloroplasts in tobacco and Arabidopsis cells. NPR1-GFP appeared as a blurry spreading image throughout the cytoplasm (Supplementary Fig. 4e). Although fluorescence from cytosolic NPR1 was contaminated with out-of-focus chloroplastic fluorescence, its intensity in the cytosol significantly increased at 3 h and 6 h after salt stress.

**Figure 5:**
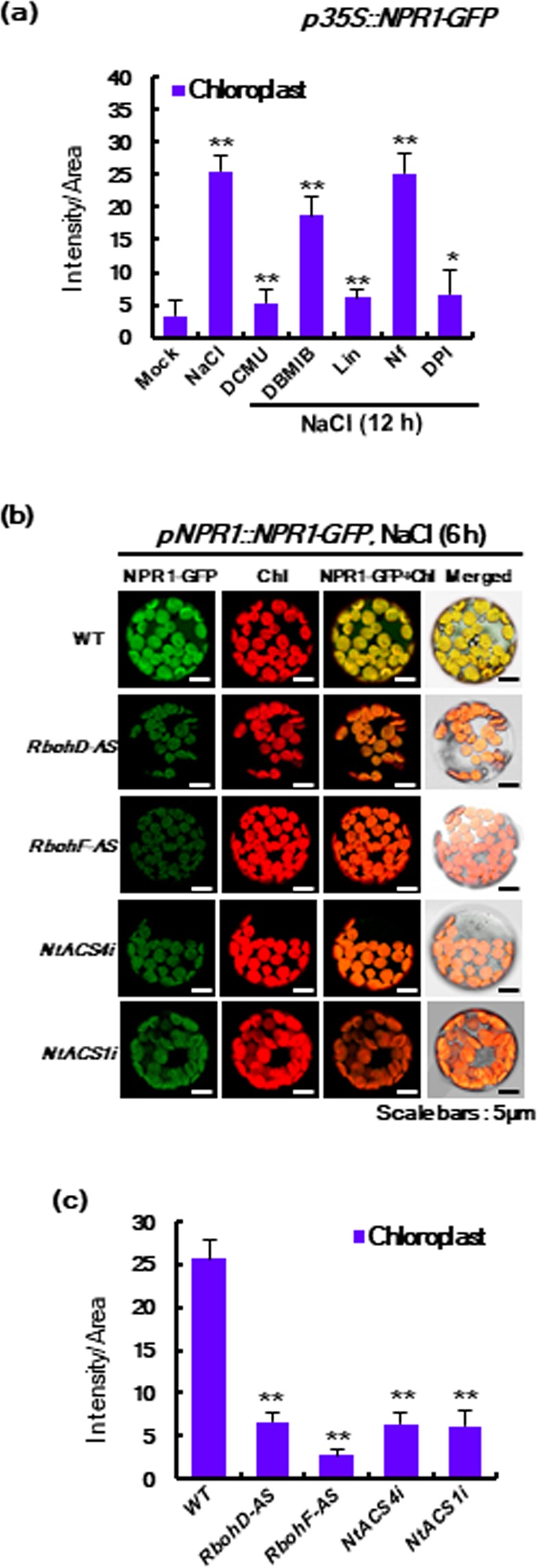
Translocation into chloroplast is dependent on stress-induced ROS and requires chloroplast functionality in response to salt stress. (**a**) Fluorescence intensity of NPR1-GFP in chloroplasts in *p35S::NPR1-GFP* transgenic plants under salt stress for 12 h. Inhibitors were co-treated with salt stress. Inhibitors: 3-(3,4-dichlorophenyl)-1,1-dimethylurea (DCMU), 2,5-dibromo-3-methyl-6-isopropylbenzoquinone (DBMIB), Lincomycin (Lin), Norflurazone (Nf), and Diphenyleneiodonium (DPI). (**b)** CLSM images of NPR1-GFP fluorescence after electroporation of *pNPR1::NPR1-GFP* construct into mesophyll protoplasts from stable transgenic tobacco plants with antisense expression lines (*RbohD-AS* and *RbohF-AS*) of NADPH oxidase genes, *RbohD* and *RbohF* and RNAi lines (*NtACS4i* and *NtACS1i*) of 1-aminocyclopropane-1-carboxylic acid (ACC) synthase genes, *ACS1* and *ACS4*. Data represent from six (**a**) or four (**b,c**) independent experiments with n = 20.

We next examined the accumulation of NPR1 in chloroplasts by altering their conditions such as redox status. Two inhibitors, 3-(3,4-dichlorophenyl)-1,1-dimethylurea (DCMU) and 2,5-dibromo-3-methyl-6-isopropylbenzoquinone (DBMIB), are known to inhibit the electron transport chain in PS II^30^. We first determined the intracellular levels of *p35S*-driven NPR1-GFP fluorescence using ImageJ. Co-treatment with DCMU and salt stress significantly reduced NPR1 accumulation in chloroplasts (Fig. 5a, supplementary Fig. 5). However, DBMIB weakly prevented translocation of NPR1 into chloroplasts. DCMU results in oxidation of plastoquinone (PQ), whereas DBMIB leads to reduction of PQ^25^. It was recently reported that DCMU inhibits chloroplastic H_2_O_2_ synthesis during stomatal closure^31^, whereas DBMIB shows the opposite effect^32^. Therefore, the results that DCMU down-regulated the accumulation of chloroplastic NPR1 suggested that DCMU-induced inhibition of H_2_O_2_ synthesis significantly reduced the transport of NPR1 into chloroplasts.

Norflurazone (Nf), a compound inducing strong photo-oxidation and subsequent plastid dysfunction^33^, showed a similar level of chloroplast NPR1 accumulation with salt treatment in leaf protoplasts (Fig. 5a), suggesting Nf-induced phytotoxicity does not interrupt the accumulation of NPR1 in chloroplasts. Next, diphenyleneiodonium (DPI), an inhibitor of NADPH oxidase^34^, was administered to leaves of *p35S::NPR1-GFP* transgenic plants. DPI-dependent inhibition of NADPH oxidase activity^34^ resulted in reduction of its products, including ROS, in chloroplasts and other cellular compartments. As expected, DPI treatment significantly reduced NPR1 accumulation in chloroplasts (Fig. 5a). These results also suggest that chloroplast NPR1 might be dependent on the oxidative status of chloroplasts.

Lincomycin (Lin), a translation initiation inhibitor in intact chloroplasts^35^, was administered to transgenic plants of NPR1-GFP. As treatment with Lin began, the fluorescence intensity of stress-induced NPR1 decreased significantly in chloroplasts (Fig. 5a). These results imply that the translocation pathway of NPR1 into chloroplasts is required for translation of proteins in chloroplasts.

ROS are major components of redox homeostasis in living organisms^36^. A synergistic effect between ROS and ethylene production in tobacco plants was previously reported^37^. Different members of 1-aminocyclopropane-1-carboxylic acid synthase (ACS) genes were involved in the biphasic phase of ethylene production: *NtACS4* in the early phase at 1–3 h and *NtACS1* in the late phase at 48–72 h after pathogen infection^38^. Therefore, we used *NtACS4*- and *NtACS1*-silenced transgenic plants (*NtACS4i* and *NtACS1i*) via RNA interference-mediated repression. Stress-induced NPR1 localization in chloroplasts was significantly reduced in transient expression of *pNPR1::NPR1-GFP* with antisense transgenic plants of NADPH oxidase genes (*RbohD* and *RbohF*) and RNAi expression of ethylene biosynthetic genes (*NtACS4i* and *NtACS1i*) (Fig. 5b). Our results suggest that NPR1 translocation into chloroplasts is triggered by production of stress-induced ROS and ethylene.

To unravel the dependence of chloroplast NPR1 accumulation on other plant hormones, we analyzed the spatial pattern of NPR1-GFP protein in leaves after treatment with several hormones. Interestingly, fluorescent NPR1 significantly accumulated in chloroplasts in response to all tested plant hormones: indole-3-acetic acid, 6-benzyladenine, gibberellin, SA, jasmonic acid (JA), and ACC as an ethylene precursor, and ABA (Supplementary Fig. 6).

**Figure 6:**
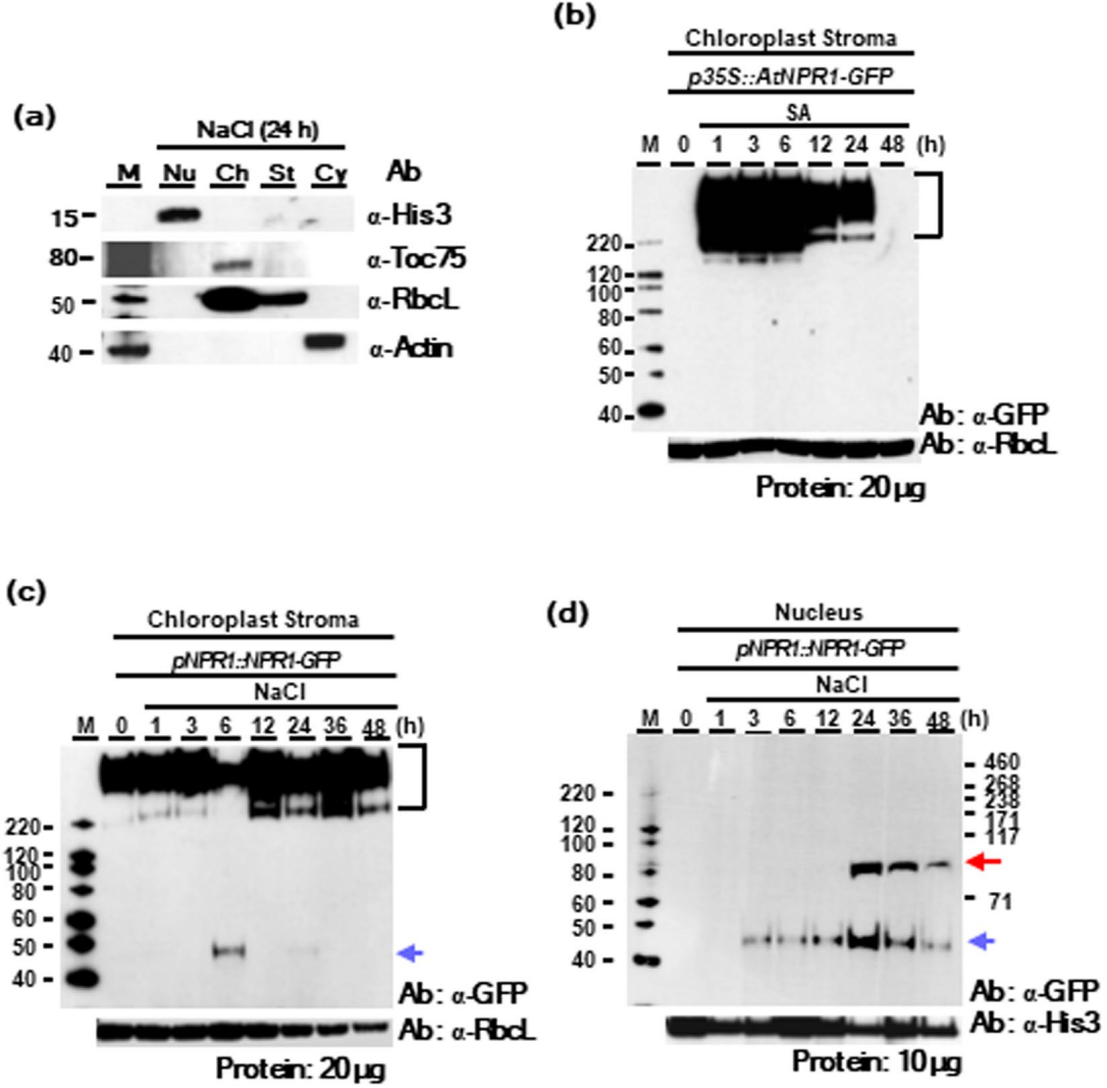
NPR1 oligomers are the main forms in chloroplasts while NPR1 monomer is the main form in the nucleus with a modified size of 45 kDa (CP45). (**a**) Western blot with subcellular fractions of the nucleus (Nu), chloroplast (Ch), stroma (St), and cytoplasm (Cy) from transgenic plants using each marker antibody. (**b)** Oligomers of AtNPR1 were present in the immunoblot analysis of the chloroplast stroma protein fraction from transient expression of *p35::AtNPR1-GFP* in mesophyll protoplasts of Arabidopsis leaves. Western blot analysis was performed by non-denatured SDS-PAGE using GFP antibody. Oligomers (square bracket). (**c**,**d)** Oligomers of NPR1s were present in the immunoblot analysis of the chloroplast stroma protein fraction from *pNPR1::NPR1-GFP* transgenic tobacco plants (**c**), and monomers with a size of 93 kDa and modified form with a size of 45 kDa were present in the nuclear protein fraction from *pNPR1::NPR1-GFP* transgenic tobacco plants (**d**) in Western blot analysis by non-denatured SDS-PAGE. Oligomers (square bracket), monomeric form (red arrow), and 45 kDa form (CP45) (blue arrow). The purity and equal loading of each fraction was proven by analyzing nuclear fraction with Histon3 antibody, and chloroplast stroma fraction with RbcL antibody. Each experiment was repeated as two (**a**), five (**b,c**), or seven (**d**) independent experiments, and after confirming that similar results were obtained, one was selected.

Next, Western blot analysis was performed using protein fractions of nuclear, chloroplast stroma, and cytoplasmic compartments. When the stroma protein fraction was analyzed using TOC75 (translocon at the outer envelope of chloroplast) antibody, no contamination with the chloroplast outer envelope was detected (Fig. 6a). Further, the purity of the fractions was confirmed by analyzing the cytoplasmic fraction with β-actin antibody, the nuclear fraction with Histon3 antibody, and the chloroplast fraction with TOC75 antibody.

NPR1-GFP oligomers with sizes of at least 200 kDa to 400 kDa (dimer to tetramer) were detected in chloroplast stroma proteins after *p35S::AtNPR1-GFP* transient expression in mesophyll Arabidopsis protoplasts (Fig. 6b) and in *pNPR1::NPR1-GFP* transgenic tobacco leaves (Fig. 6c). The oligomeric proteins larger than the dimer was significantly increased in chloroplast stroma of Arabidopsis at 1 h and 3 h, decreased from 6 h, and then completely disappeared after 48 h under SA treatment (Fig. 6b).

Especially, new molecules of about 45 kDa were observed together in protein fractions from chloroplast stroma of *pNPR1::NPR1-GFP* transgenic tobacco plants at 6 h upon salt stress, and these molecules were named CP45 (Fig. 6c). Monomers with a size of 93 kDa were detected from 24 h while molecules with a size of 45 kDa were detected from 3 h using GFP antibody in the nucleus of stressed *pNPR1::NPR1-GFP* transgenic plants (Fig. 6d). These results are in well agreement with the spatial and temporal fluorescent patterns of NPR1-GFP and indicate sequestration of NPR1 protein into both chloroplasts and the nucleus in tobacco leaves. Therefore, the existence of 45 kDa molecules of NPR1-GFP in both chloroplasts and nucleus after salt stress suggests that this molecule can move from chloroplasts to the nucleus after post-translational modification. However, whether or not the actual sequestration of NPR1 from chloroplasts to the nucleus occurred should be further investigated.

Only a small portion of the total chloroplast proteome, which lacks chloroplast transit peptide (cTP), is nucleus-encoded, and thus enters internal chloroplast compartments^39^. Some non-cTP chloroplast proteins can be localized to the stroma through the ER-dependent chloroplast targeting pathway^40^. Although tobacco NPR1 does not possess cTP and signal peptide, fluorescence of NPR1-GFP was clearly detected in chloroplast stroma, as evidenced by orange color in the merged image with chlorophyll autofluorescence (Fig. 4a,d). The positive control using GFP only was not detected in chloroplasts (Supplementary 4a), whereas the oligomeric forms of NPR1-GFP level were clearly detected in chloroplasts using western blot analysis and determination of GFP fluorescence.

NPR1 translocates to the nucleus where it interacts with TGA transcription factors^14^. Therefore, NPR1, as a transcriptional partner, stimulates the DNA-binding activities of TGA^34^ and induces pathogenesis-related (PR) gene expression in plant immune responses^41,42^. We next investigated the efficacy of nuclear-localized NPR1 as a transcriptional activator. Transcription of TGA2, an NPR1-dependent tobacco orthologue of TGA transcription factor15^43^, was effectively induced in response to salt stress and peaked at 12 h in *NPR1-Ox* (Supplementary Fig. 7). This was accompanied by enhanced expression of nuclear-encoded *PR* genes such as *PR-1*, *PR-3*, *PR-4*, and *PR-5* as SA marker genes in the later stage at 12–24 h as well as photosynthetic genes during the entire period in *NPR1-Ox* compared to WT (Supplementary Figs. 1, 2, 7), resulting in attenuation of photosynthetic loss, alleviation of cell damage, and stress tolerance.

### NPR1 protein exhibits chaperone function in chloroplasts and cytoplasm

Abiotic stresses including salt stress generally lead to accumulation of oxidized proteins and protein aggregation^22,44^, followed by interruption of protein homeostasis. Molecular chaperones are important components enhancing to the homeostasis and the quality control of proteins under stress conditions^22,45,46^. Chaperone proteins exist as multimeric structures consisting of oligomeric components^30^. Tobacco NPR1s are also prominently shown to form tetramers and even higher oligomer complexes ranging in size from about 200 kDa to larger than 400 kDa in chloroplasts in Arabidopsis (Fig. 6b) and tobacco (Fig. 6c), although NPR1 was observed in monomeric form and as a smaller protein of 45 kDa in the nucleus (Fig. 6c,d). Therefore, we investigated whether or not NPR1 has a chaperone function. Heat-induced aggregates of malate dehydrogenase (MDH) were used as a substrate to determine the chaperone activity of NPR1-GFP^30^, which was isolated by immunoprecipitation with GFP antibody from the chloroplasts, cytoplasm, and nucleus of *p35S::NPR1-GFP* transgenic plants under salt stress.

**Figure 7:**
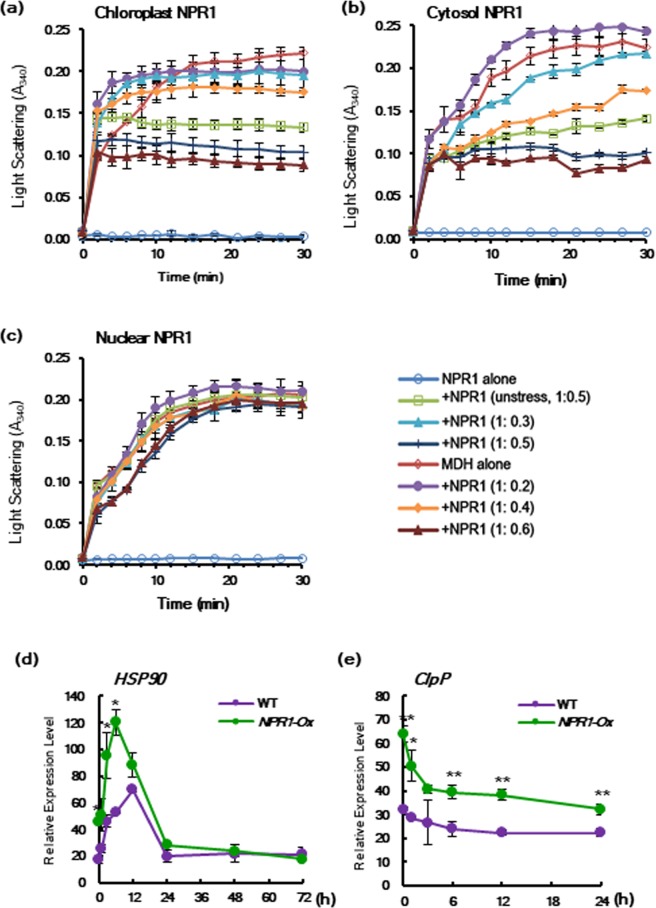
Chloroplast NPR1-GFP exhibits molecular chaperone activity *in vitro*. (**a–c**) Effects of increasing amounts of immunoprecipitated NPR1-GFP proteins on heat-mediated MDH aggregation. NPR1-GFP was prepared by immunoprecipitation from chloroplasts (**a**), cytoplasm (**b**), and nucleus (**c**) of *p35S::NPR1-GFP* transgenic plants under salt stress. NPR1-GFP was also isolated from unstressed control transgenic plants. NPR1-GFP proteins were determined using a model substrate MDH (0.5 μM) under thermal denaturing conditions (45 °C) for 30 min at various molar ratios. (**d**,**e)** Transcription levels of heat shock protein 90 (HSP90) (**d**) and caseinolytic protease subunit P (ClpP) (**e**) in *NPR1-Ox* and WT after salt stress. Each experiment was repeated as four (**a–c**) or three (**d,e**) independent experiments with n = 3, and after confirming that similar results were obtained, one was selected.

MDH protein at 45 °C showed substantial aggregation after 10 min, whereas purified NPR1 proteins alone from chloroplasts and cytoplasm did not form any aggregates. When purified NPR1 was added to MDH, marked reduction in light scattering was observed, indicating NPR1 prevented heat-induced aggregation of substrate (Fig. 7a–c). As the ratio of NPR1 to MDH increased, more amount of MDH remained soluble while MDH aggregates decreased, indicating NPR1 indeed functions as a molecular chaperone. At an MDH:NPR1 molar ratio of 1:0.6, the strongest chaperone activity was observed. On the other hand, NPR1 purified from unstressed transgenic plants showed a reduced chaperone activity of stressed NPR1 by 23%, suggesting the NPR1 forms under salt-stressed conditions were inherently different from NPR1 forms under unstressed conditions.

Cytosolic NPR1 purified from *p35S::NPR1-GFP* transgenic plants under salt stress showed chaperone activity similar to that of chloroplast NPR1 (Fig. 7b). In contrast, nuclear NPR1 purified from the same transgenic plants under salt stress did not show chaperone activity for any of the tested molar ratios of MDH:NPR1 (Fig. 7c). Taken together, these results demonstrate that NPR1 proteins in chloroplasts and the cytoplasm function effectively as molecular chaperones during salt stress.

We next investigated whether or not overexpression of NPR1 has effects on other molecular chaperones under salt stress. Heat shock protein 90 (HSP90) chaperone is an essential regulator of proteostasis in eukaryotic cells^47^. Transcription of HSP90 was rapidly elevated after salt stress and reached a peak at 12 h in WT (Fig. 7d). However, the transcription level of HSP90 was significantly and rapidly increased in *NPR1-Ox* transgenic plants after salt stress compared with WT. Therefore, it is suggested that NPR1 can induce other molecular chaperones in addition to being active.

The ATP-dependent Clp protease is highly conserved in the chloroplasts^48^ and mitochondria of eukaryotic cells^49^. Chloroplast-encoded ClpP is a core subunit of Clp protease, which is the most abundant stromal protease in chloroplasts^48^. In the unstressed control, the transcription level of *ClpP* was 2-fold higher in *NPR1-Ox* transgenic compared with WT leaves, indicating NPR1 contributes to plastid protein homeostasis (Fig. 7e).

### NPR1 might play roles in redox homeostasis in chloroplasts

**Figure 8:**
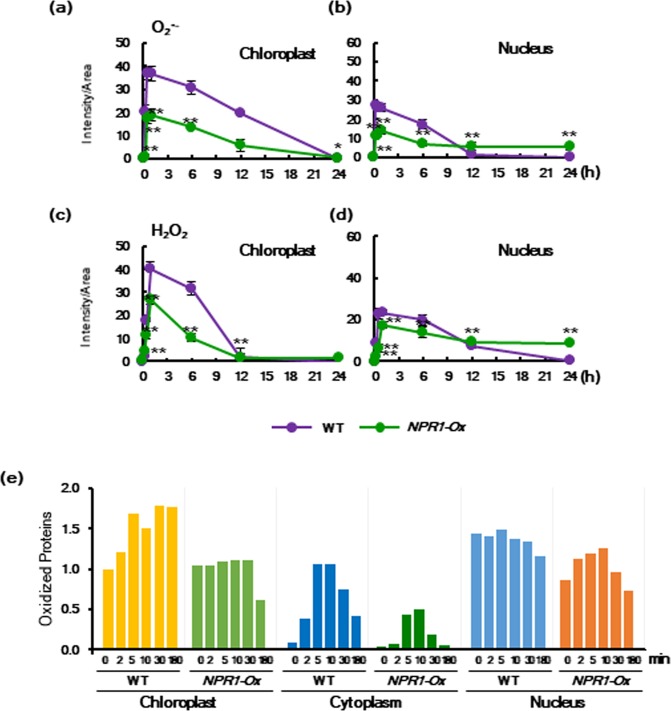
Overexpression of NPR1 transiently reduce levels of ROS and oxidized proteins. (**a–d**) Kinetics of ROS production in response to salt stress. ROS accumulation in mesophyll protoplasts was measured using a CLSM after staining with BES-So for superoxide anion (**a**,**b**) and BES-H_2_O_2_ for hydrogen peroxide (**c**,**d**). (**e)** Overexpression of NPR1 reduced amounts of stress-induced oxidized proteins in chloroplasts, cytoplasm, and nucleus. Levels of oxidized proteins were quantified in WT and *NPR1-Ox* transgenic plants by Oxyblot analysis. Proteins from chloroplasts, cytoplasm, and nucleus were extracted from salt-stressed leaves and then derivatized by DNP, followed by immunoblotting with anti-DNP antibodies. DNP signals were quantified by ImageJ with the WT control value set as 1. Each experiment was repeated as five independent experiments with n = 20 (**a–d**) or single treatment (**e**), and after confirming that similar results were obtained, one was selected.

To investigate further physiological significances of chloroplast NPR1 in response to salt stress, we determined ROS levels in WT and *NPR1-Ox* transgenic plants under salt stress. Stress-induced intracellular levels of superoxide anion and hydrogen peroxide were investigated using the highly specific dyes benzene sulfonyl (BES)-So and BES-H_2_O_2_, respectively^22^. Stress-induced ROS levels were significantly inhibited in chloroplasts of *NPR1-Ox* compared to WT, suggesting that ROS generation might be closely related to the localization of NPR1 in chloroplasts (Fig. 8a–d). The inhibitory effects of NPR1 overexpression on chloroplast and nuclear accumulation of ROS were relatively prominent during the early stages. However, the inhibitory effects of NPR1 overexpression on the accumulation of ROS in both organelles were ineffective after 12 h, when the NPR1 level was significantly reduced in the chloroplasts. Even superoxide anion and H_2_O_2_ in the nucleus were detected at slightly higher levels in *NPR1-Ox* than WT.

Therefore, it is questionable whether or not sequestration of NPR1 to the nucleus actually has a function for ROS detoxification under salt stress. NPR1 overexpression considerably up-regulated transcription of ROS-detoxifying enzymes such as superoxide dismutase (MnSOD and CuZnSOD), catalase, and glutathione-S-transferase phi^50^ during relatively late stage of the stress period (Supplementary Fig. 8). However, NPR1 overexpression up-regulated transcription of cytosolic ascorbate peroxidase (APXc) during the whole period of salt stress. These results imply that intracellular accumulation of stress-induced ROS was significantly reduced by NPR1 function as a transcription coactivator for genetic reprogramming at a relatively later stage of salt stress.

Normally, ROS-induced translocation of signaling molecules and transcription factors to the nucleus enhances the transcript levels of protective antioxidant enzymes^51^. It has been recently reported that concentration-dependent regulation by ROS is essential in signaling pathways for stress resistance^52^, and thus the concentration levels of ROS are a determinant of cell viability^52^. The dual nature of ROS, either beneficial or detrimental, underscores the need to identify thresholds that determine which direction will act in the cells^52^. In this study, we observed that plant cells under salt stress rapidly up-regulated a redox-sensitive NPR1 protein, which translocated to chloroplasts for induction of protective responses while lowering chloroplastic ROS accumulation (Fig. 8a).

To investigate the effects of stress-induced NPR1 on the cellular redox state, we determined oxidative status in *NPR1-Ox* and WT plants under salt stress. The levels of oxidized proteins were investigated using an OxyBlot protein oxidation detection kit (Merck Millipore, USA)^22^. In WT leaves, oxidized proteins in chloroplasts accumulated immediately from 2 min after salt stress, reaching a peak at 5 min and then maintaining a high level until 3 h (Fig. 8e). However, the amount of chloroplast-oxidized proteins did not increase in *NPR1-Ox* transgenic plants after salt stress and was instead maintained at control level until 30 min. On the other hand, the levels of oxidized proteins were reduced after 3 h. Taken together, chloroplast NPR1 has positive effects by directly lowering stress-induced oxidized proteins (Fig. 8e), which is related with lower levels of ROS accumulation (Fig. 8a–d).

Compared with chloroplasts, the levels of oxidized proteins in the cytoplasm were significantly lower in WT and *NPR1-Ox* during stress. In WT, salt stress induced a transient increase in the accumulation of oxidized proteins from 2 min and a final peak at 5 min (Fig. 8e, third panel). After 10 min, cytoplasmic levels of oxidized proteins gradually decreased. In *NPR1-Ox*, the pattern of transient accumulation in cytoplasmic-oxidized proteins was similar with that of WT, whereas the levels of oxidized proteins were significantly lower. NPR1 overexpression also induced the reduction of oxidized protein levels in the nucleus, even though its effect is somewhat weaker in the nucleus than in chloroplasts and the cytoplasm (Fig. 8e, fifth and sixth panel).

## Discussion

If light is not properly utilized for photosynthesis in chloroplasts, it will not only change the redox state and produce ROS but will also damage the photosynthetic machinery^53^. Therefore, chloroplasts that absorb light have to act as environmental sensors, and they communicate with the nucleus about the conditions of abiotic/biotic stress and even development.

Since ROS represent a common response product to almost all environmental stresses in plants, they could be the primary trigger of the signaling pathway, followed by the redox-triggered cascade response. Our finding of chloroplast localization of transcription co-activator NPR1 as a direct primary response against ROS-induced oxidative damage demonstrates the possibility of chloroplast-to-nucleus communication during salt stress. Furthermore, the intracellular localization-dependent functions of NPR1 in chloroplasts may coordinate protein homeostasis and gene expression during salt stress.

In unstressed cells, Arabidopsis NPR1 is predominantly localized to the cytoplasm as a tetrameric complex^15^. Upon exposure to pathogen infection or biotic stressors such as SA, JA, and ABA, alteration of the cellular redox state is triggered, thereby reducing NPR1 tetramer into a monomer, after which NPR1 monomer is imported into the nucleus to function as a coactivator of gene transcription for activating defense-related downstream genes^18^. Surprisingly, we identified novel functions for NPR1 as a chaperone and an antioxidant in chloroplasts upon salt stress using stable transgenic plants with native *NPR1* promoter-driven *NPR1-GFP* and transient expression of *35* *S* promoter-driven *AtNPR1-GFP* constructs.

Under salt stress, NPR1 mRNA and protein were immediately *de novo* synthesized (Fig. 1a) and then rapidly translocated into chloroplasts (Fig. 4a,d), where it was shown to function directly as a molecular chaperone (Fig. 7a) and antioxidant (Fig. 8e). Stress-induced accumulation of NPR1 protein, which does not contain the transit peptide, was detected in chloroplasts, where it was mainly present in multimeric form in Arabidopsis and tobacco (Fig. 6b,c). Levels of oxidized proteins immediately increased in chloroplasts of WT after salt stress, whereas chloroplasts of *NPR1-Ox* transgenic plants did not show any increase (Fig. 8e). In addition, overexpression of NPR1 significantly reduced the amount of stress-induced oxidized protein in the cytoplasm compared with WT. We also found that overexpression of NPR1 governs a more sensitive state of the redox-regulated clock (Fig. 3a,c), which is more advantageous for plants with increasing photosynthetic adaptability in response to adverse environmental conditions. Taken together, stress-induced chloroplast NPR1 may play dual roles as both a redox regulator and molecular chaperone in tobacco plants.

It has been well illustrated that NPR1 plays a role as a master immune regulator upon SA-mediated translocation from the cytoplasm to nucleus in plants^54^. It was recently reported that the NPR1-dependent SA signaling pathway is pivotal for enhanced tolerance against salt and oxidative stress in Arabidopsis^55^. Our work is the first report showing that NPR1 is immediately involved in stress resistance like a first aid for sustaining photosynthetic capability in chloroplasts. NPR1 with dual functions in the chloroplasts is very effective against high salinity.

A large population of proteins is oxidized upon abiotic/biotic stress, and these proteins aggregate and become cross-linked, potentially forming toxic species^56^. To prevent this, NPR1 is rapidly imported into chloroplasts to avoid aggregation of oxidized proteins and promotes efficient degradation of proteins. Especially, NPR1 proteins from the chloroplast fraction of *NPR1-Ox* under salt stress showed more effective chaperone activity compared with that in unstressed plants (Fig. 7a), suggesting NPR1 is an efficient molecular chaperone whose activity is regulated in a stress-dependent manner^30^. The chaperone activity of NPR1 is responsible for decreases in the levels of oxidized proteins in chloroplasts of *NPR1-Ox* transgenic plants (Fig. 8e), implying protein quality control and maintenance of proteome homeostasis are important for NPR1-induced stress tolerance.

The hydrophobic structure of NPR1 may contribute to protein stability and polymeric form in chloroplasts, and its function was shown to be related with chaperone activity in a stress-dependent manner (Fig. 7a). NPR1 was annotated with the molecular function of protein-binding by analysis using database InterPro (https://www.ebi.ac.uk/interpro/)^57^, including not only transcription factor-binding and protein dimerization activity but also binding to unfolding proteins and chaperone. The main reason why NPR1 has been shown to represent these functions in the InterPro database is that NPR1 has several Ankyrin repeats, which are degenerate 33–amino acid repeats that serve as domains for protein–protein interactions^58^. Proteins with ankyrin repeats have been reported to act as chaperones. Therefore, it is possible that chaperone activity of NPR1 was mediated by binding to a chaperone or by direct chaperone function.

Proteostasis is achieved by an integrated network of numerous proteins, including molecular chaperones^46^. Proteostasis mediated through the chaperone activity of chloroplast NPR1 was shown to be related to stress tolerance of enhanced chloroplast gene expression, cell viability, and photosynthetic capability with maximal quantum yield of PS II (Fig. 1b–d). In fact, *NPR1-Ox* transgenic plants under unstressed control conditions were taller, their leaves were a little bit larger and darker green, and their mean biomass of one leaf from 6-week-old plants was 26 mg FW/m^2^. This biomass is 30% higher than that of WT, although any other phenotype did not change.

Our observations imply that NPR1-induced changes are related to chaperone activity for proteostasis in the cytoplasm and chloroplasts and maintained redox homeostasis in intracellular compartments. These effects are initiated by stress-induced redox changes through ROS production, the level of which may be properly regulated under threshold level to avoid any harmful effects. Although stress-induced ROS plays a positive role in stress signaling, it is reduced in *NPR1-Ox* relative to WT (Fig. 8a), suggesting that the regulation of ROS to an appropriate level by NPR1 seems to be more beneficial at increasing stress resistance.

More interestingly, our results imply that NPR1 has different functions in chloroplasts and the nucleus. The switch of NPR1 function is related to the intracellular redox status originating from environmental conditions, indicating that NPR1 can serve as a communicator and provide emergency restoration after sensing stress-induced redox status. The original dogma that every polypeptide fulfils only one function has been replaced by the notion that many are bi- or even multifunctional^53^. One strategy for a protein from one gene to exhibit dual or multiple functions is to import into different intracellular compartments by spatial or temporal pattern. NPR1 protein is synthesized *de novo* in response to salt stress and is translocated into a primary compartment (chloroplast), after which it enters a secondary compartment (nucleus). Our studies have demonstrated this possibility and NPR1 should be further studied since it has been shown to participate in signaling movement as well as have dual functions in chloroplasts and the nucleus.

## Methods

### Plant materials and growth conditions

Surface-sterilized seeds of tobacco (*Nicotiana tabacum* L. Wisconsin 38) plants were grown on solid Murashige and Skoog (MS) medium (pH 5.8) under light (16 L/8D, 100 μmol photons m^−2^ s^−1^) at room temperature (25 ± 5 °C), which were followed by the previously reported experimental method^17^. Fully-matured WT and T1, T2, or T3 plants after antibiotic selection were subjected to salt stress (200 mM NaCl) or salicylic acid (SA) treatment (300 μM). Solutions with salt, SA, and other chemicals were applied to whole leaves with petiole in 20 mM MES buffer under light (100 μM photons m^−1^s^−1^) at 25 °C. For mock treatment, tobacco leaves or whole plants were floated on MES buffer without any chemicals^17^.

### Fluorescent fusion constructs and transgenic plants

For *p35S::AtNPR1-GFP*, the full-length of open reading frame (ORF) of At*NPR1* (GenBank: ATU76707) was PCR-amplified and the resulting product cloned into *pMBP* vector harboring *35* *S* promoter-driven green fluorescence protein (GFP) gene and *NOS terminator*. For *p35S::NPR1-GFP*, the open reading frame (ORF) of *NPR1* (GenBank: KY402167) was PCR-amplified and the resulting product cloned into *pMBP* vector harboring *35* *S* promoter-driven green fluorescence protein (GFP) gene and *NOS terminator*. In control transgenic plants with free GFP, pMBP vector with *35* *S* promoter-driven *GFP* gene alone and *NOS terminator* was used as a transgenic construct. PCR-amplified full-length of *NPR1* was overexpressed by cloning into *pMBP* vector harboring *35S* promoter and *NOS* terminator after removal of the GFP gene, which was named *NPR1*-*Ox* construct. Further, native *NPR1* promoter from genomic DNA of *Nicotiana tabacum* was amplified by PCR, after which the DNA fragment was gel-purified using a QIA quick Extraction Kit (Quigen). The 0.8 kbp DNA fragment of *NPR1* promoter was cloned into promoter-less *NPR1-GFP* construct, which was prepared from *p35S::NPR1-GFP* after deletion of *35S promoter* fragment. Resulting constructs were introduced into *N. tabacum* by *Agrobacterium* (strain LBA 4404)-mediated transformation. Successfully transformed T1 plants were kanamycin-resistant and then selected for further propagation. Lines with T2 progeny segregating 3:1 for kanamycin resistance:sensitivity were further propagated, and homozygous T3 plants were used for further study in the cases of *NPR1-Ox* and *p35S::NPR1-GFP*. In the experiments with *pNPR1::NPR1-GFP* transgenic plants, T1 plants were used after selection of kanamycin resistance. Surface-sterilized transgenic seeds were cultured on solid Murashige and Skoog medium (pH 5.8) under light (16 L/8D, 100 μmol photons m^−2^ s^−1^) at room temperature (25 ± 5 °C)^22^.

### RNA isolation and real-time qPCR

The isolation of Total RNA was performed as previously described^17^. To analyze transcription levels by real-time qPCR, 1 μg of total RNA from leaves was reverse-transcribed for 30 min at 42 °C in a 20 μl of reaction volume using a High Fidelity PrimeScriptTM RT-PCR kit (Takara, Japan) according to the manufacturer’s instructions. Gene-specific PCR primers for qPCR were designed according to a stringent set of criteria (Supplementary Table 1), including a predicted melting temperature of 60 °C ± 5 °C, primer lengths of 20 to 24 nucleotides, guanine-cytosine content of 50 to 60%, and PCR amplicon lengths of 100 to 250 bp. Sequence information for PCR primer was obtained from the GenBank database. Real-time qPCR was performed in Thermal Cycler Dice^®^ Real Time System III T950 (Takara, Japan). A 20 μl of reactions consisted of 10 μl of 2X SYBR Green Master Mix, 0.5 μM of each primer, and 10 ng of cDNA. PCR conditions were as follows: 95 °C for 15 min, 45 cycles of 95 °C for 30 s, 57 °C for 30 s, and 72 °C for 30 s, extended by 72 °C for 10 min. Fluorescence threshold data (Ct) were analyzed using Thermal Cycler Dice^®^ Real Time System III Software version 6.0 (Takara, Japan) and then exported to Microsoft Excel for further analysis. Relative transcription levels in each cDNA sample were normalized to the reference gene β-actin after qPCR. The mean relative mRNA expression level for each gene in WT and overexpressing transgenic plants (*NPR1-Ox*) was obtained, and the expression ratio for each gene between WT and *Ox* plants was calculated^17^.

Nucleus-encoded genes: *Rbc S*, RubisCO Small subunit; *PsaF*, Photosystem I reaction center subunit III; *PsaK*, Photosystem I subunit X; *PsaN*, Photosystem I reaction center subunit XII; *CAB*, Chlorophyll a/b-binding protein; *CAB3*, Chlorophyll a/b-binding protein 3; *CAB21*, Chlorophyll a/b-binding protein 21; *HSP90*, heat shock protein 90; *CAB36*, Chlorophyll a/b-binding protein 36; *LHCB6*, Photosystem II light-harvesting chlorophyll-binding protein CP24.

Chloroplast-encoded genes: *Rbc L*, RubisCO Large subunit; *PsbA*, Photosystem II reaction center D1 protein; *PsbN*, Photosystem II subunit; *PsaB*, Photosystem I reaction center; *ClpP*, Caseinolytic protease subunit P; *PsaJ*, Photosystem I subunit IX.

### Trypan blue staining

This experiment was performed as previously described^59^. To determine plant cell death, salt-treated tobacco leaves were immersed for 1 min in a boiling solution consisting of 10 ml of lactic acid, 10 ml of glycerol, 10 g of phenol, and 0.4% (w/v) trypan blue. After plants had cooled to room temperature, solution was replaced with 70% (w/v) chloral hydrate. Stained plants were decolorized overnight to removing chlorophyll and then imaged using a digital camera.

### Analysis of photosynthetic activity

Photosynthetic activity was measured as previously described^37^. Steady-state net photosynthesis of eight-week-old whole plants was determined using a Gas Exchange Measuring Station (Walz, Germany) with a built-in light source (210 *μ*mol photons m^−2^ s^−1^). Gas stream (60 l h^−1^, 21% O_2_, 430 μl^−1^ CO_2_) was provided continuously into the measuring chamber by a mass-flow control system. Leaves were maintained at 25 °C and 70 ± 1% humidity conditions.

### Detection of GFP localization

Transgenic expression of NPR1-GFP in leaves, roots, and protoplasts prepared from transgenic NPR1-GFP tobacco plants. For free GFP expression, control transgenic plants with *35* *S::GFP* were used. Protoplasts were prepared by incubation in enzyme solution (0.5 M mannitol, 1 mM CaCl_2_, 20 mM MES, 0.1% BSA, 1% cellulase R-10, and 0.25% marcerozyme R-10)^22^. GFP measurements were performed as previously described^22^. GFP fluorescence in cells was detected using a confocal laser scanning microscope (FluoView 300, OLYMPUS, Japan) or a fluorescence microscope (DM4000 BLED, Leica, Germany) equipped with a high resolution CCD camera. GFP expression was visualized by excitation at 488 nm and emission at 520 nm. Chlorophyll fluorescence was visualized by excitation at 458 nm and emission at 647–720 nm. Fluorescence of DAPI (4′,6-Diamidino-2-Phenylindole) staining for nuclei was visualized by excitation at 358 nm and emission at 461 nm. Fluorescence density was quantified with ImageJ bundle software provided by the Wright Cell Image facility.

### Confocal microscopy detection of ROS in protoplasts

For fluorescent detection of ROS, leaf epidermal strips were used. Leaf epidermal strips were peeled from tobacco leaves with salt stress after the indicated time. Leaf epidermal strips were floated on the indicated solution. BES-So and BES-H_2_O_2_ (WAKO Chemicals, Japan) are fluorescent probes for superoxide and hydrogen peroxide, respectively^59^. BES-H_2_O_2_ was used at a concentration of 50 mM in 20 mM potassium phosphate buffer (pH 6) for 1 h in the dark (excitation, 485 nm; emission, 530 nm). BES-So was used at a concentration of 20 mM potassium phosphate buffer (pH 6) for 1 h in the dark (excitation, 505 nm; emission, 544 nm). Fluorescence was observed using a confocal laser scanning microscope FluoView 300 (FV 300; Olympus).

### Chloroplast and nuclear isolation, protein extraction, and western blotting

To extract total protein from tobacco leaves, frozen samples were ground into powder and suspended in protein extraction buffer (50 mM Tris-HCl, pH 7.5, 150 mM NaCl, 5 mM EDTA, 0.1% Triton X-100, 0.2% Nonidet P-40 (NP-40) containing 50 μg/ml of tosyl-L-phenylalaninyl-chloromethylketone, 50 μg/ml of tosyl-L-lysine-chloromethylketone, serine protease inhibitors, 0.6 mM phenylmethylsulfonyl fluoride (PMSF), 80 μM MG115, 80 μM MG132, and complete protease inhibitor cocktail tablet (Roche, USA)^20^.

To isolate chloroplast stroma proteins from tobacco leaves, chloroplasts were first isolated from leaves using a chloroplast isolation kit (Sigma-Aldrich, USA), after which intact chloroplasts were harvested by 40/80% Percoll gradient. Intact chloroplasts were suspended in chloroplast lysis buffer (0.5 mM HEPES-KOH, pH 7.5, 2 mM MgCl_2_, 1 mM NaF, 1 mM EDTA, 1 mM PMSF, 80 μM MG115, 80 μM MG132, 10 μM pepstatin A and one complete protease inhibitor cocktail tablet (Roche, USA)). After lysate centrifugation, supernatants were recovered as total proteins or chloroplast stroma proteins. Inhibition of proteasome-dependent degradation was accomplished by 40 μM MG115.

To isolate nuclear proteins from tobacco leaves, nuclei were first isolated from leaves, after which nuclear proteins were isolated using a plant nuclei isolation/extraction kit CelLytic^TM^ PN (Sigma-Aldrich, USA). According the manufacturer’s protocol, nuclei were collected from leaves with nuclei isolation buffer by mesh filtering. Cell lysate was prepared with 2.3 M sucrose by centrifugation at 12,000 × g for 10 min, after which the supernatant was discarded. The nuclei pellet was then added to nuclear protein extraction buffer. Nuclei proteins were then added to Working Extraction Buffer in addition with 80 μM MG115 and 80 μM MG132 and then centrifuged for 10 min at 12,000 × g. Pure supernatant was used to obtain nuclear proteins.

To extract cytosolic proteins from tobacco leaves, fresh leaves were homogenized with homogenization buffer (50 mM HEPES-KOH, pH 7.5, 250 mM sorbitol, 50 mM potassium acetate, 2 mM magnesium acetate, 1 mM EDTA, 1 mM EGTA, and 1 mM DTT, 80 μM MG115, 80 μM MG132, and one complete protease inhibitor cocktail tablet (Roche, USA)). After removal of cell debris by centrifugation for 10 min at 500 × g, sequential centrifugations of the supernatant at higher speeds were performed. Crude nuclear and chloroplast fractions were removed at 1,000 × g for 15 min; mitochondrial fraction was removed at 20,000 × g for 15 min; final supernatant was harvested to obtain the cytosolic proteins.

Proteins (100 μg of total proteins, 20 μg of chloroplast stroma proteins, 10 μg or 20 μg of nuclear proteins, or 20 μg of leaf cytosolic proteins) were separated by 4–12% Bis-Tris Plus (Novex, USA). Western blotting was performed using a Mini gel tank and Mini Blot Module (Life Technologies, USA) according to the manufacturer’s protocol. Proteins were transferred onto iBlot 2 NC Regular Stacks (Novex, Israel), after which blots were blocked using iBind Cards (Novex, Israel) according to the manufacturer’s instructions. NPR1-GFP proteins were detected by reacting with mouse monoclonal anti-GFP monoclonal antibody (Clontech, USA) and horseradish peroxidase-conjugated secondary antibody (Santa Cruz, USA). Bands were visualized using SuperSingnal West Substrate Working Solution (Thermo Scientific, USA) on X-ray film. To identify TOC proteins in the chloroplast outer envelope membrane, polyclonal rabbit antibody of Toc75–3 (*Pisum sativum* Toc 75 POTRA domain 3) (Agrisera, Sweden) was used. We employed primary antibodies for RubisCO protein, ubiquitin, and cellular proteins. Other primary antibodies were as follows: anti-RbcL (Agrisera), anti-His3 (Agrisera), and anti-actin (Agrisera).

### Oxidized protein analysis

Oxidized proteins were detected using an OxyBlot protein oxidation detection kit (Merck Millipore, USA) according to the manufacturer’s protocols^22^. Dinitrophenylhydrazine (DNP) was added to samples to derive carbonyl groups from the protein side chains. Derivatized samples were separated by electrophoresis, as described above. Western blot analysis was performed using 2,4-DNP antibody (1:150). DNP signals in integrated intensity of each lane were quantified by densitometry using ImageJ software and normalized to the total protein value of the WT 0 h control, which was set as 1.

### Transient expression in tobacco protoplasts

Mesophyll protoplasts from tobacco leaves or Arabidopsis WT leaves were isolated using protoplast extract enzyme solution (pH 5.7) consisting of 1% cellulose R-10 and 0.25% marcerozyme R-10. Leaf slices were transferred to a Petri dish containing enzyme solution and then incubated in the dark for 12 h at 25 °C. After incubation, the enzyme solution was discarded by mesh (10 mm) filtration, after which the cells were overlaid with 1 ml of W5 buffer (154 mM NaCl; 5 mM KCl; 125 mM CaCl_2_; 5 mM glucose; 1.5 M MES, pH 5.7)^60^. After gentle centrifugation (5 min at 80 g), protoplasts floating at the interface were collected, washed with W5 (3/1 v/v), pelleted by centrifugation (10 min at 80 g), and resuspended in W5 solution. After stabilization of protoplasts in ice for 30 min, protoplasts at a density of 10^6^/ml were used for further transient transformation.

Protoplasts (300 µl) in W5 buffer were pipetted gently into a 0.4 cm pre-chilled electroporation cuvette, and 50 µg of DNA constructs in 10 µl of TE buffer was added. Electroporation was performed using the Gene Pulser Xcell System (BIORAD, Hercules, CA, USA). Electroporation was carried out with 160 V/960 μF (voltage/capacitance), according to the manufacturer’s instructions. After electroporation, the cuvette was chilled on ice for 10 min, after which protoplasts were transferred to a conical tube using a glass Pasteur pipette with addition of 500 μl of K3 media (154 mM NaCl; 125 mM CaCl_2_; 5 mM sucrose; 5 mM xylose; 1.5 mM MES, pH 5.7). These protoplasts were monitored with a confocal microscope.

### Immunoprecipitation using anti-GFP antibody

For the immunoprecipitation of GFP-fused NPR1 proteins, chloroplast stroma proteins and nuclear proteins were separately extracted from *p35S::NPR1-GFP* transgenic tobacco leaves with immunoprecipitation buffer (1X phosphate-buffered saline, pH 7.4 (Cat. no. 10010–031, ThermoFisher Scientific, USA)) containing MG115, MG132, and plant protease inhibitor cocktail (Sigma, USA). Protein lysates (30 µg) were precleared with 50 µl of sheep anti-rabbit magnetic beads in a microcentrifuge tube at room temperature for 1 h with gentle rotation. To the precleared lysate, 5% NGS (Normal Goat Serum, pH 7.4) in PBS was added for blocking, after which primary anti-GFP antibody diluted in PBS was added to a final concentration of 0.2 µg/ml. After incubation of the mixture at 4 °C overnight with gentle rotation, the supernatant was discarded, and the bead mixture was washed in wash buffer (5% NGS in PBS, 1% Triton® X-100, 3% BSA) by pipetting gently up and down. Bound proteins were eluted by boiling in 25 µl of 1X SDS sample buffer. The supernatant was analyzed by SDS-PAGE and was used as a substrate for chaperone activity.

### Analysis of chaperone activity

The chaperone activity of NPR1-GFP was assayed by measuring its capacity to suppress thermal aggregation of malate dehydrogenase (MDH) from malic dehydrogenase from porcine heart (Sigma-Aldrich, Saint Louis, MO, USA) as a model substrate^22,30^. MDH was incubated in 50 mM Hepes-KOH (pH 8.0) buffer with various molar ratios of NPR1-GFP recombinant protein purified by immunoprecipitation with GFP antibody from transgenic plants with *p35S::NPR1-GFP* after salt stress. Aggregation of the substrate was determined under heat denaturation at 45 °C for 30 min by measuring the turbidity at 340 nm using a Shimadzu UV-1601 spectrophotometer (Shimadzu, Japan).

### Quantitation and statistical analysis

All experiments were repeated at least three times with three replicates, and data from one representative experiment are presented^61^. Statistically significant differences according to t-test between transgenic lines and respective controls at each time point are indicated with one asterisk (*) (P < 0.05) or two asterisks (**) (P < 0.01). Two-way ANOVA was also performed to investigate statistical differences between responses of WT and transgenic lines.

## Supplementary information

Supplementary information.
