# Supplementary material for: Functional switching of NPR1 between chloroplast and nucleus for adaptive response to salt stress
Source: Sci Rep. 2020 Mar 9;10:4339. doi: 10.1038/s41598-020-61379-3 (PMC7062895; doi:10.1038/s41598-020-61379-3)

Title Page

Title: Functional switching of NPR1 between chloroplast and nucleus for adaptive response to salt stress

Authors: So Yeon Seo, Soo Jin Wi, Ky Young Park

- Corresponding author: Ky Young Park

Affiliation: Department of Biology, Sunchon National University, Sunchon, Chonnam, Republic of Korea

Table S1. Sequences of primers for real-time qRT-PCR

Gene	Strand	Sequences
β-Actin	Forward<br>Reverse	TCACAGAAGCTCCTCCTAATCC<br>GGGAAAGAACAGCCTGAATG
NPR1	Forward<br>Reverse	GCTGTAGCATATTGCGATGC<br>GCAACATGCAGCACTGTGTA
Rbc L	Forward<br>Reverse	ACCTGAGTACCAAACCAAGGA<br>TCAAGGCTGGTAAGTCCATC
PsbA	Forward<br>Reverse	TGCTGTTGCATATTCAGCTCC<br>CCTAACATGTGAAATGGGTGC
PsbN	Forward<br>Reverse	ATGGAAACAGCAACCCTAGTC<br>CTAGTCTCCATGTTCCTCCAA
PsaB	Forward<br>Reverse	ATTTGATGGGCTGGTTAAGGG<br>CAATTCCTGCCAATATCCACG
PsaJ	Forward<br>Reverse	AACATATCTCTCTGTGGCACC<br>AAAGGGGAATGTCAACGCATC
Chl I	Forward<br>Reverse	AGCTCCAGAACCAAATCGAC<br>TCTTGCTGCCCTGTTAGTGA
ClpP	Forward<br>Reverse	TTAGGCCAAGAGGTTGATAGC<br>ACATCTGGTCGCACAAATTGC
Rbc S	Forward<br>Reverse	AGGTGTGGCCACCAATTAAC<br>GTCTCGAATTCCAAGCAAGG
CAB3	Forward<br>Reverse	ATTGGGTCTTGCTGAAGATCC<br>TTCCGGGAACAAAGTTTGTGG
CAB13	Forward<br>Reverse	GCAATGCTTGGAGCACTAGG<br>CTGAGCATGCACAAGGTTAGG
CAB21	Forward<br>Reverse	GTCCATTCTCCGGTGAGTCC<br>CACATCCAAGAGCACCAAGC
CAB36	Forward<br>Reverse	CATTCGCTAGGAACCGTGAG<br>TATCTGAGATCCGGCCTTGA
LHCB6	Forward<br>Reverse	AAGGTGGGTGGACTTCTTCA<br>GAATTTGCCACCAGGGTAAC
PsaF	Forward<br>Reverse	CCATGCAAGGAGTCTAAGCA<br>GCAATCCATCTGATCCACAC
PsaK	Forward<br>Reverse	ATGAGGCGTAAAGGACAAGG<br>GCCTTCCTATTTGCTGATGG
PsaN	Forward<br>Reverse	TTGGTGCATCTGAACTCACC<br>GCTTGGTTCTTGTTGGCTTC

HSP90	Forward<br>Reverse	TATCCAAGACATGTCCCAGTG<br>CAGATGTGGCAGATGAAGTAG
TGA2	Forward<br>Reverse	ACAAGCAGAAGCCCATTTGG<br>TGGCCTTGAACTCCAATTGG
PR-1	Forward<br>Reverse	TTGAGATGTGGGTCGATGAG<br>CCTAGCACATCCAACACGAA
PR-3	Forward<br>Reverse	AGGAACGACGGTAGATGTCC<br>TCCTACGGGCAGTATCATCA
PR-4	Forward<br>Reverse	ATGGCTGGACTGCTTTCTGT<br>CTCACTGTTGCTTGAGTTCCTG
PR-5	Forward<br>Reverse	GTCGTAATCTCAGATGCACAGC<br>AGTAGGCCCACATGATCCAG
MnSODmi	Forward<br>Reverse	GGAGGTCACATTAACCACTCG<br>CAGCACCTTCTGCATTCATC
CuZnSODc	Forward<br>Reverse	CATGGTGCTCCTGAAGATGAG<br>GATTGTGGACCAGCAAGAGG
APXc	Forward<br>Reverse	TGTTCCCTTTCACCCTGGTAGAG<br>CGTTCCTTGTGGCACCTTCC
Catalase1	Forward<br>Reverse	CGCCATGCTGAGAAGTATCC<br>AAAGCGTTCTTGCCTGTCTG
Catalase2	Forward<br>Reverse	CTACGATTCGATTGCTGCTG<br>GGCCAGGTCTTGGTTACATC
GSTF	Forward<br>Reverse	CGAAGCGCAATTGTCTAAGG<br>ACACCATGCACTCACACGAG

Supplementary Fig. 1

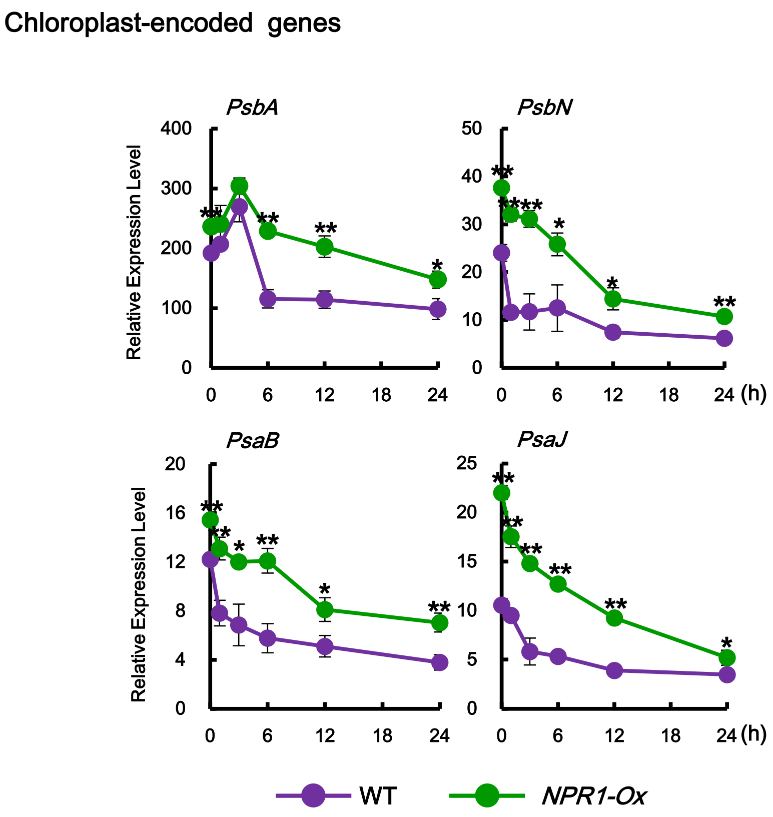

Supplementary Fig. 1. Kinetics of chloroplast-encoded gene transcription involved in photosynthesis upon salt stress.

Transcription levels were measured by real-time qPCR in response to salt stress induced by 200 mM NaCl in WT and *NPR1-Ox* transgenic plants. Transcription levels were expressed relative to that of the reference gene β-actin after qRT-PCR. Chloroplast-encoded genes: *PsbA*, Photosystem II reaction center D1 protein; *PsbN*, Photosystem II subunit; *PsaB*, Photosystem I reaction center; *PsaJ*, Photosystem I subunit IX. Relative mRNA expression levels were expressed as means ± SD. An asterisk indicates a significant difference between WT and transgenic plants with stress-treated or untreated cases (one asterisk ($P < 0.05$) or two asterisks ($P < 0.01$)).

Supplementary Fig. 2

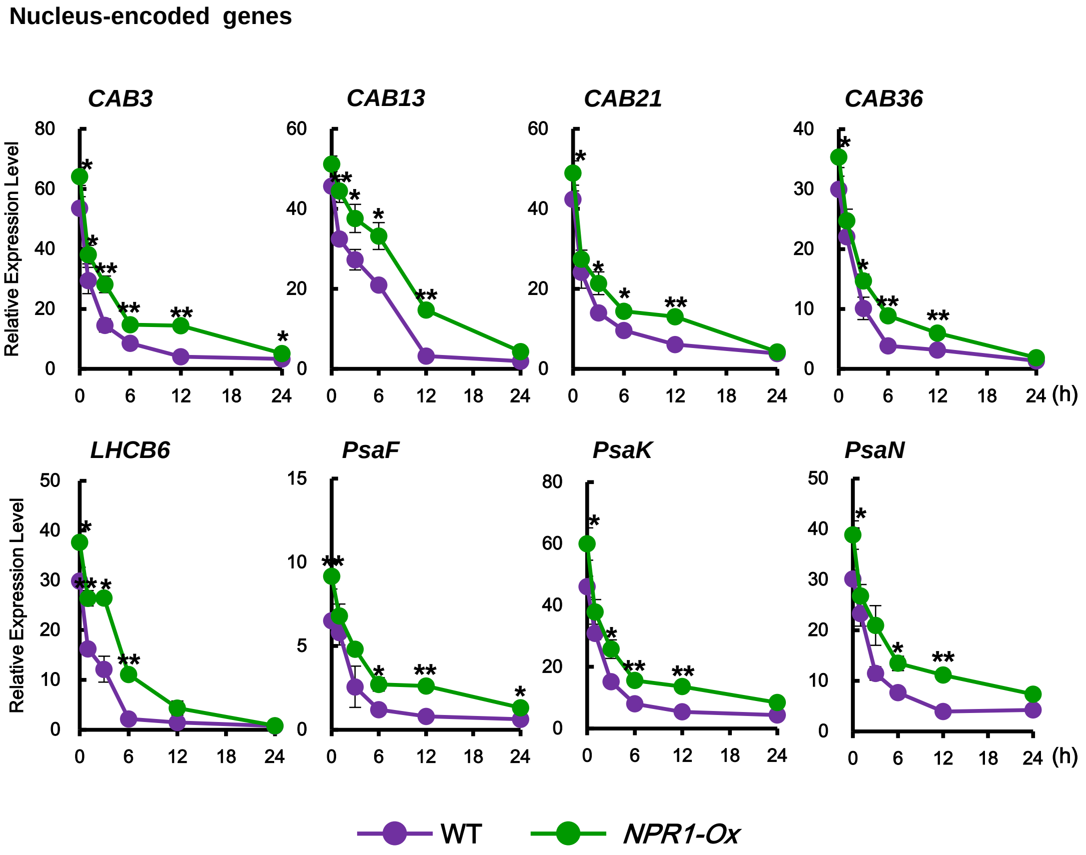

Supplementary Fig. 2. Kinetics of nucleus-encoded gene transcription involved in photosynthesis upon salt stress.

Transcription levels were measured by real-time qPCR in response to salt stress induced by 200 mM NaCl in leaves of WT and *NPR1-Ox* transgenic plants. Transcription levels were expressed relative to that of the reference gene β-actin after qPCR. Nucleus-encoded genes: *CAB3*, Chlorophyll a/b-binding protein subunit 3; *CAB13*, Chlorophyll a/b-binding protein subunit 13; *CAB21*, Chlorophyll a/b-binding protein subunit 21; *CAB36*, Chlorophyll a/b-binding protein subunit 36; *LHCB6*, Photosystem II light-harvesting complex protein 6; *PsaF*, Photosystem I reaction center subunit III; *PsaK*, Photosystem I subunit X; *PsaN*, Photosystem I reaction center subunit XII. Relative mRNA expression levels were expressed as means ± SD. An asterisk indicates a significant difference between WT and transgenic plants with stress-treated or untreated cases (one asterisk ($P < 0.05$) or two asterisks ($P < 0.01$)).

Supplementary Fig. 3

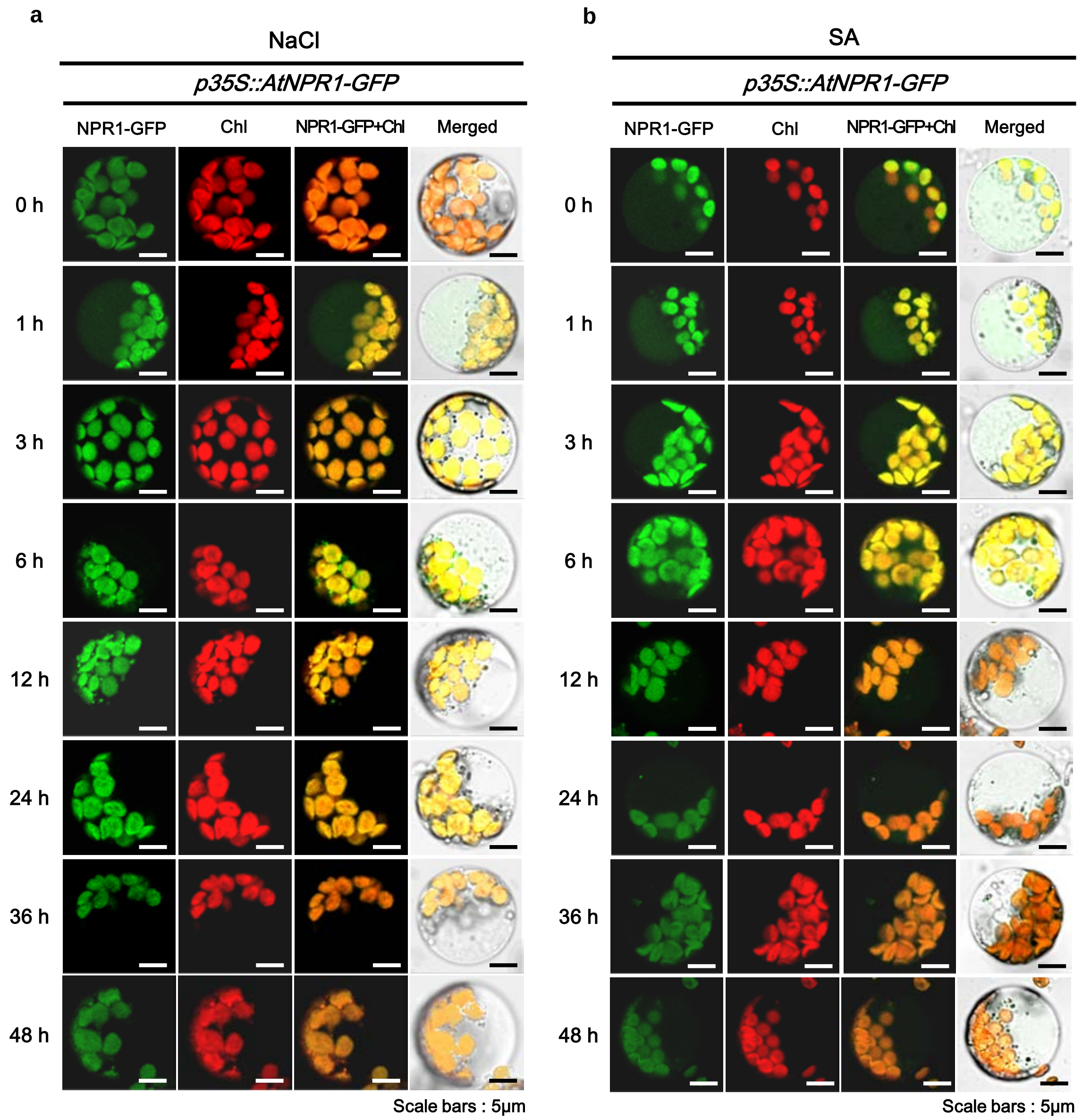

Supplementary Fig. 3. Arabidopsis NPR1 (AtNPR1) is localized to chloroplasts of Arabidopsis.

Localization of GFP fluorescence in cellular compartments of *p35S::AtNPR1-GFP* transient expression in mesophyll protoplasts of Arabidopsis WT leaves of 6-week-old seedlings. CLSM images of GFP fluorescence (green) were observed until 48 h after salt stress (**a**) and SA treatment (**b**). Microscopic image of GFP fluorescence (green) and chlorophyll autofluorescence from chloroplasts (red) are merged in the third column. Scale bars = 5 μm.

Supplementary Fig. 4

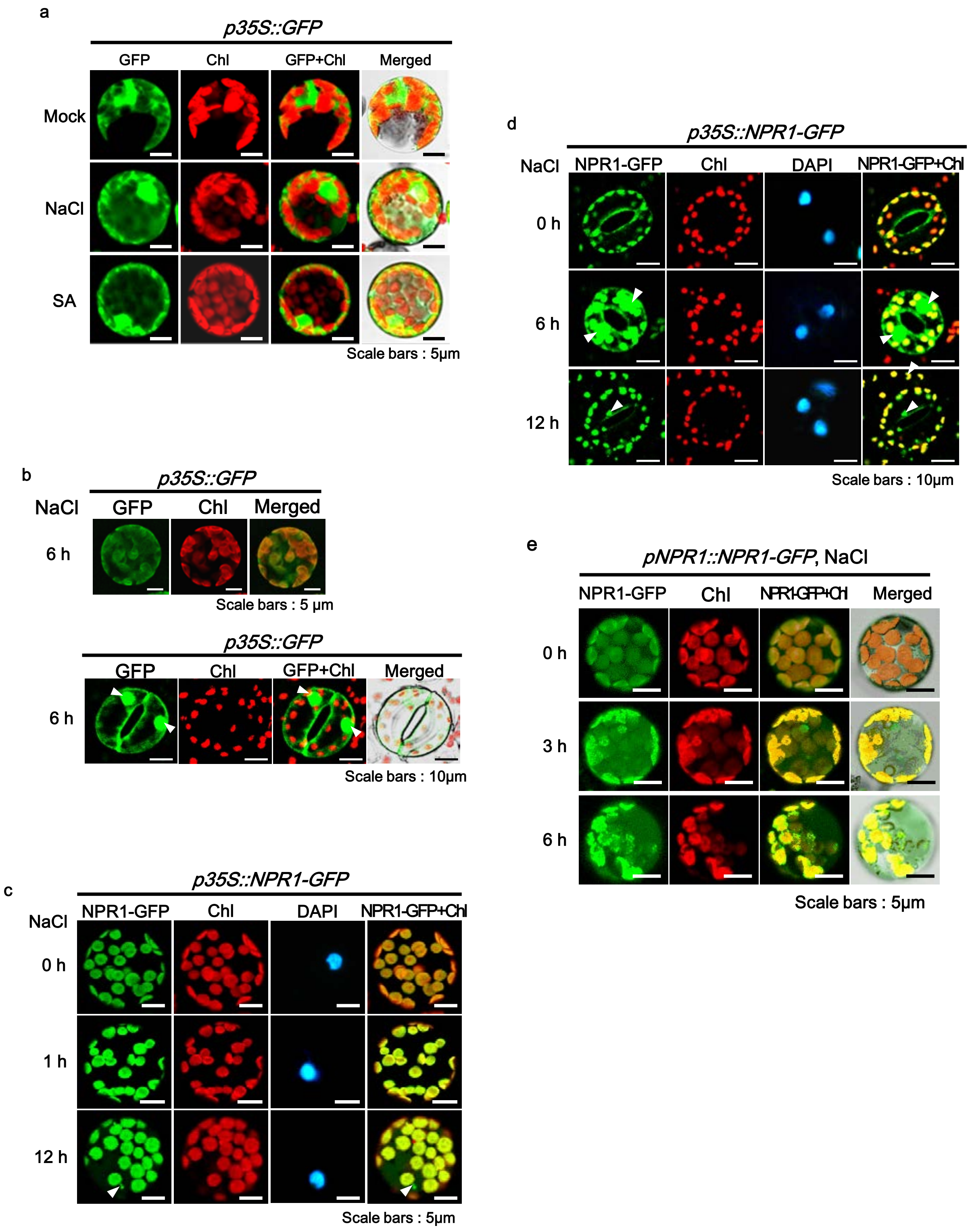

Supplementary Fig. 4. NPR1 is localized to chloroplasts and the nucleus of mesophyll cells and guard cells in Arabidopsis and tobacco leaves under salt stress.

a Expression of control vector (*p35*-driven GFP) led to GFP fluorescence in the plasma membrane, cytoplasm, and nucleus of mesophyll protoplasts of Arabidopsis leaves after salt stress with 100 mM NaCl (upper panel) and SA treatment (lower panel) with weak or no expression in chloroplasts. **b** Expression of control vector (*p35*-driven GFP) led to GFP fluorescence in the plasma membrane, cytoplasm, and nucleus of mesophyll cells (upper panel) and guard cells (lower panel) with weak or no expression in chloroplasts of mesophyll cells of tobacco leaves. **c**-**d** CLSM images of GFP fluorescence (green) were observed using leaves of 6-week-old seedlings after salt stress in mesophyll protoplasts (**c**) and guard cells (**d**) from *p35S::NPR1-GFP* transgenic plants until 12 h of salt stress. CLSM images of GFP fluorescence (green), chlorophyll autofluorescence from chloroplasts (red), and blue staining of DAPI are merged in the fourth column. **e** CLSM images of NPR1-GFP fluorescence in mesophyll protoplasts were photographed with a focus on cytoplasm. Fluorescence images of GFP were photographed in protoplasts after isolation from 6-week-old stable transgenic plants of *pNPR1::NPR1-GFP*. Scale bars = 5 μm (protoplasts) or 10 μm (guard cells).

Supplementary Fig. 5

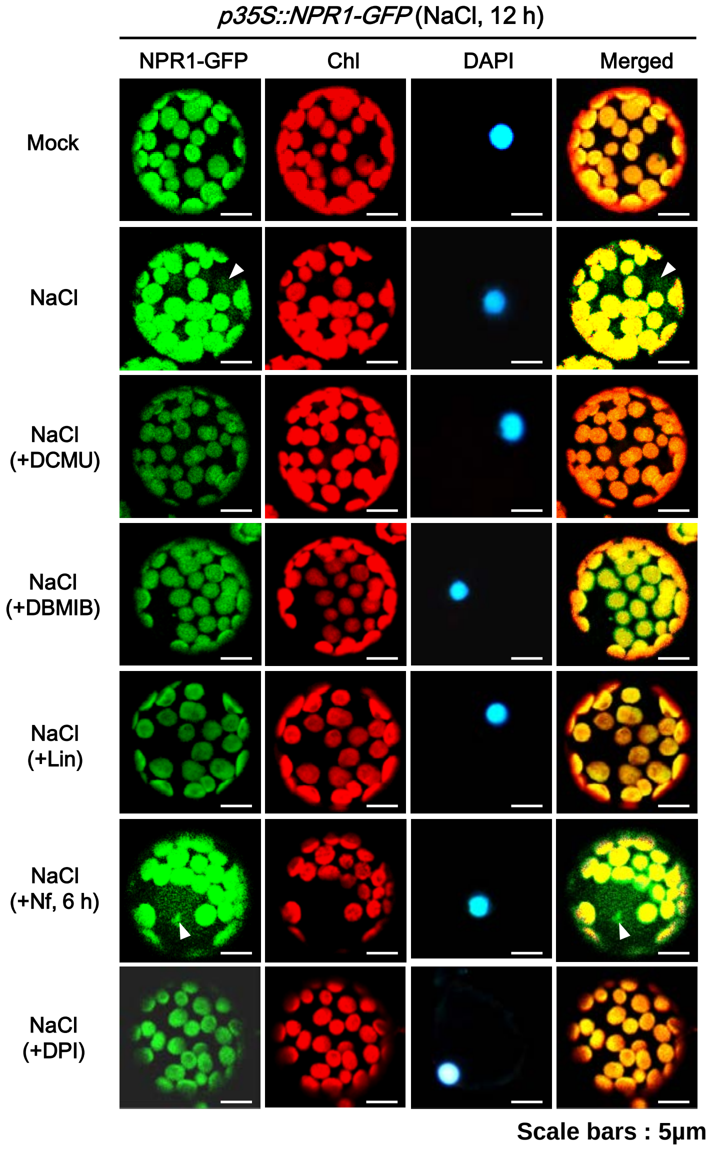

Supplementary Fig. 5. Localization of NPR1-GFP in addition to some inhibitors.

Localization of NPR1-GFP to cellular compartments of *p35S::NPR1-GFP* transgenic plants after treatments with photosynthetic inhibitors (DCMU and DBMIB), inhibitor of prokaryotic protein synthesis (Lin), inhibitor of carotene synthesis (Nf), and inhibitor of NADPH oxidase (DPI) upon salt stress. CLSM images of GFP fluorescence (green) were observed in leaves of 6-week-old seedlings at 6 h or 12 h after salt stress. CLSM images of GFP fluorescence (green), chlorophyll autofluorescence from chloroplasts (red), and blue image of DAPI staining are merged in the fourth column. Scale bars = 5 μm.

Supplementary Fig. 6

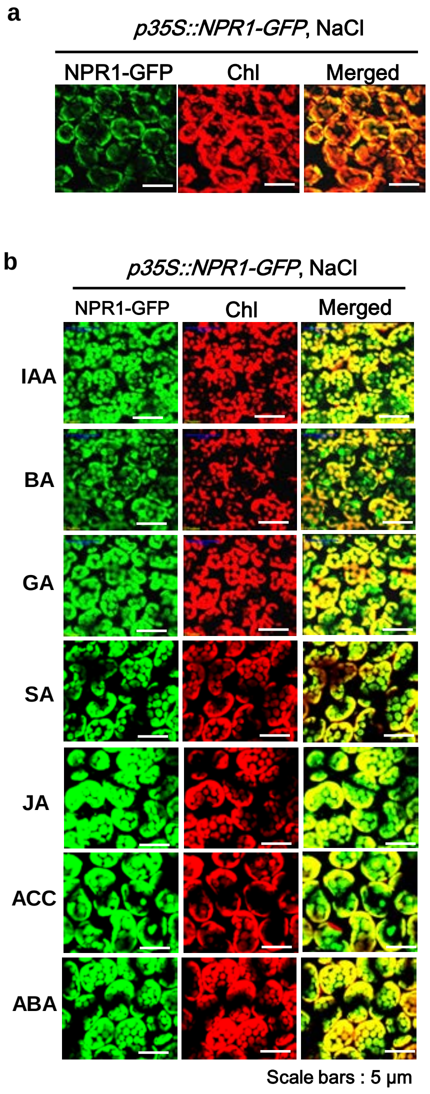

Supplementary Fig. 6. Amounts of chloroplast NPR1 are elevated in response to several plant hormones

CLSM images of GFP fluorescence (green) were observed in mesophyll cells of 6-week-old transgenic plants (*p35S::NPR1-GFP*) without (**a**) and with 12 h of plant hormone treatment (**b**), as follows: auxin (indole-3-acetic acid, IAA), cytokinin (benzyl adenine, BA), gibberellins (GA), salicylic acid (SA), jasmonic acid (*JA*), 1-aminocyclopropane-1-carboxylic acid (*ACC, precursor of* ethylene), and abscisic acid (ABA). Confocal microscopic image of GFP fluorescence (green) and chlorophyll autofluorescence from chloroplasts (red) are merged in the third column. NPR1-GFP signals overlap nicely with chlorophyll autofluorescence, suggesting NPR1-GFP translocated into chloroplasts. Scale bars = 5 μm.

Supplementary Fig. 7

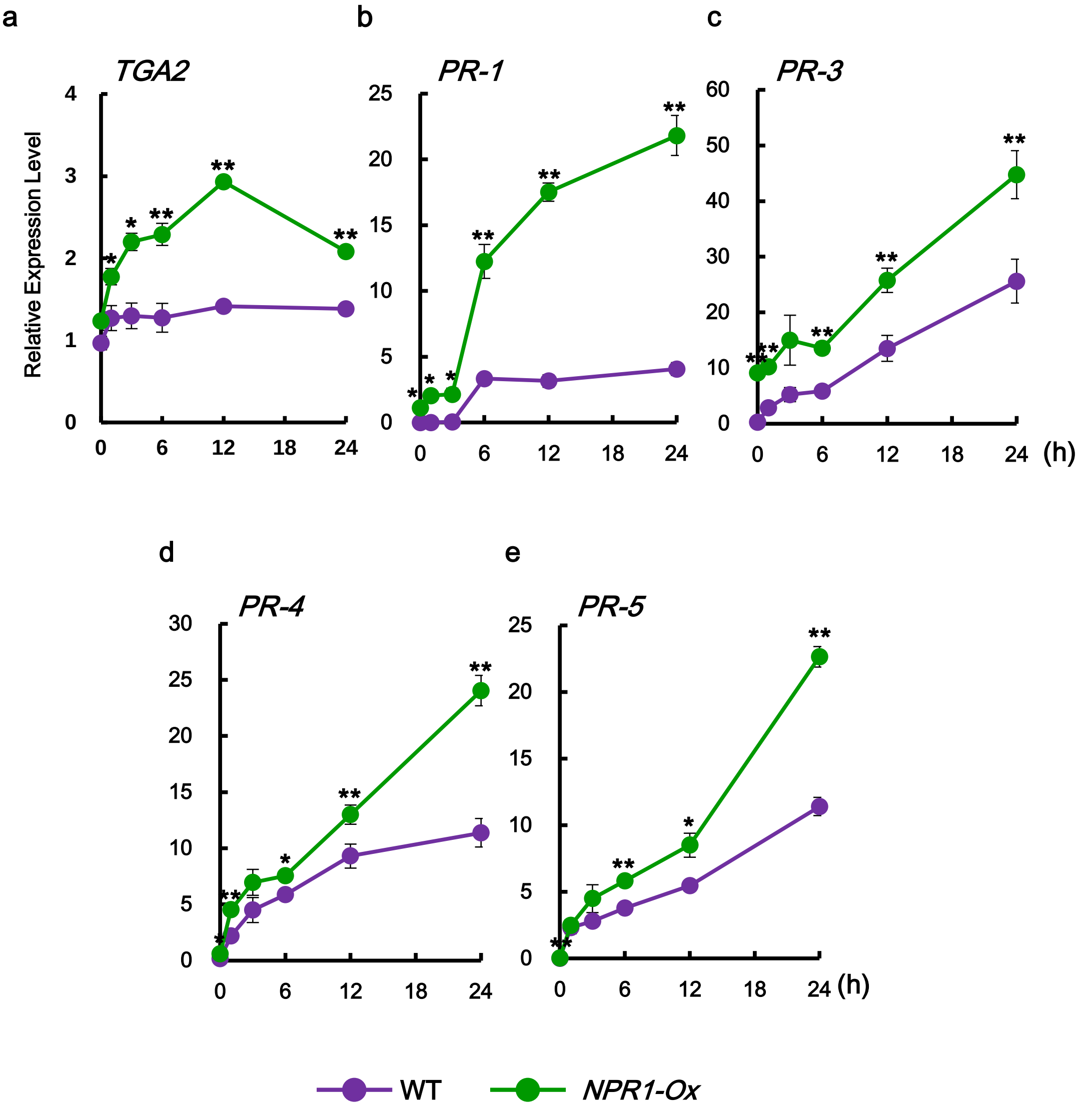

Supplementary Fig. 7. Overexpression results in enhanced transcription of *TGA2* and pathogenesis-related (*PR*) genes under salt stress.

Transcription levels of TGA2 (**a**), PR-1 (**b**), PR-3 (**c**), PR-4 (**d**), and PR-5 (**e**) were expressed relative to the reference gene β-actin after real-time qRT-PCR. Relative mRNA expression levels were expressed as means ± SD. An asterisk indicates a significant difference between WT and transgenic plants under stress-treated or untreated conditions (one asterisk ($P < 0.05$) or two asterisks ($P < 0.01$)).

Supplementary Fig. 8

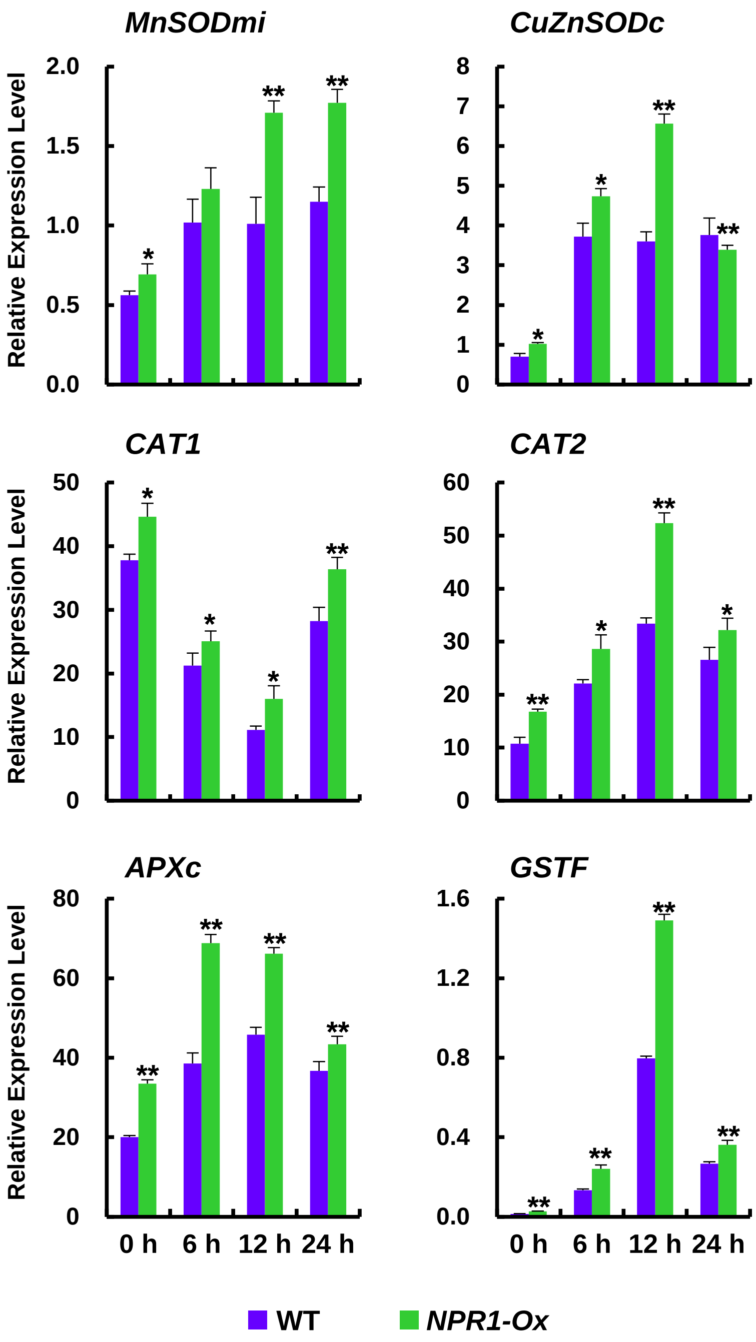

Supplementary Fig. 8. Kinetics of ROS detoxification gene transcription in response to salt stress.

Transcript accumulation of endogenous ROS detoxification enzymes, MnSODmi, CuZnSODc, CAT1, CAT2, APXc, and GSTF, in response to salt stress. Transcription levels were expressed relative to that of the reference gene β-actin after qPCR. Relative mRNA expression levels were expressed as means ± SD. An asterisk indicates a significant difference between WT and transgenic plants under stress-treated or untreated conditions (one asterisk ($P < 0.05$) or two asterisks ($P < 0.01$)).

Supplementary Fig. 9

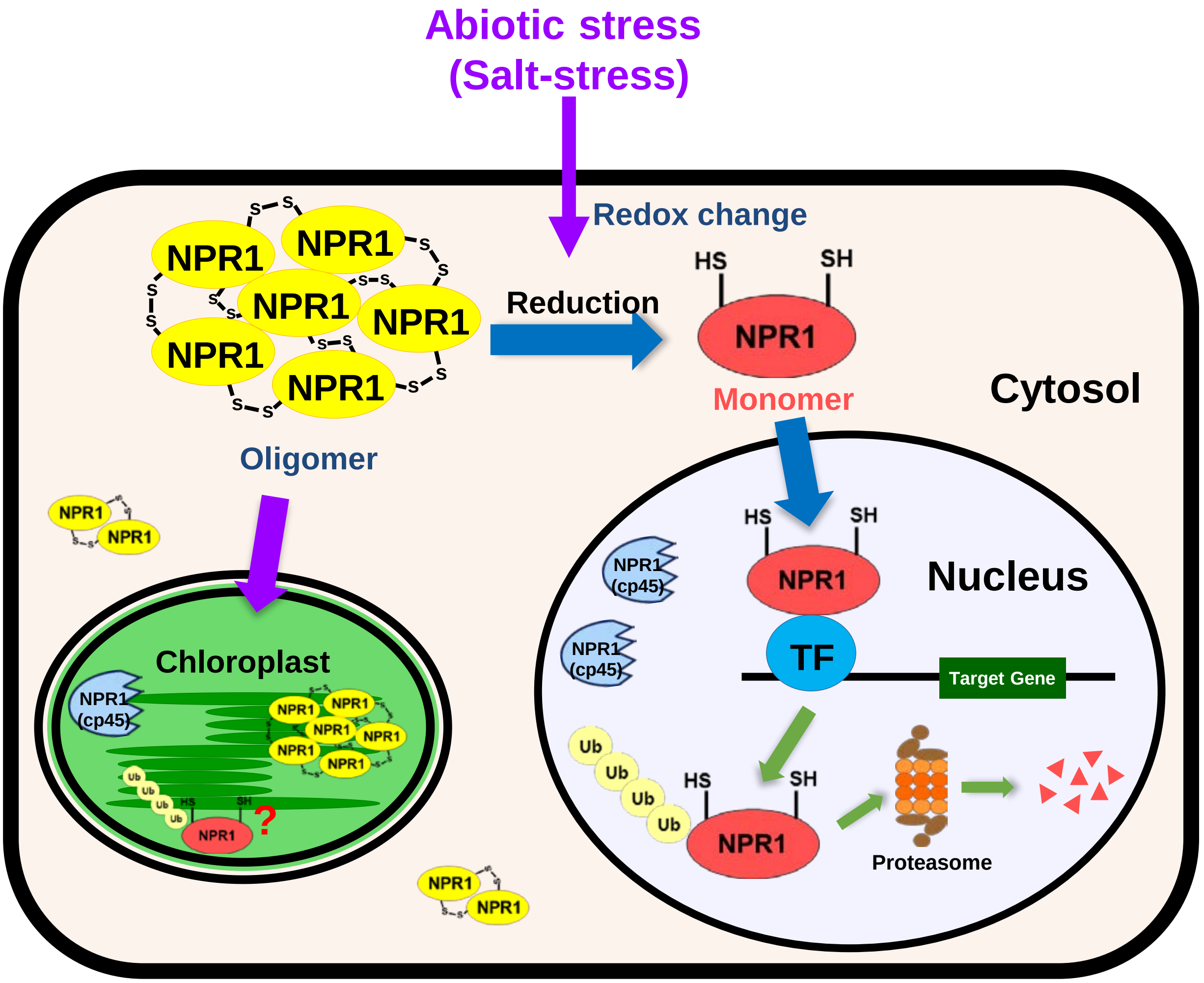

Supplementary Fig. 9. Model for dual signaling of NPR1 into chloroplasts and the nucleus under salt stress.

ROS-dependent signaling of NPR1 was accompanied by dual translocation into chloroplasts (purple arrows) and the nucleus (blue arrows) in response to salt stress. Stress-induced translocation of NPR1 oligomers larger than tetramer size into chloroplasts was observed, followed by nuclear localization of monomeric form.

The full-length blots of Fig. 6a

First Row: Western blot with α-Histon3

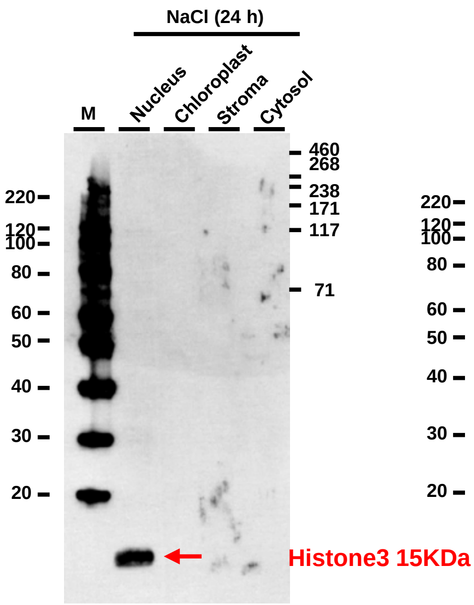

Ab: α-His3

Second Row: Western blot with α-Toc75

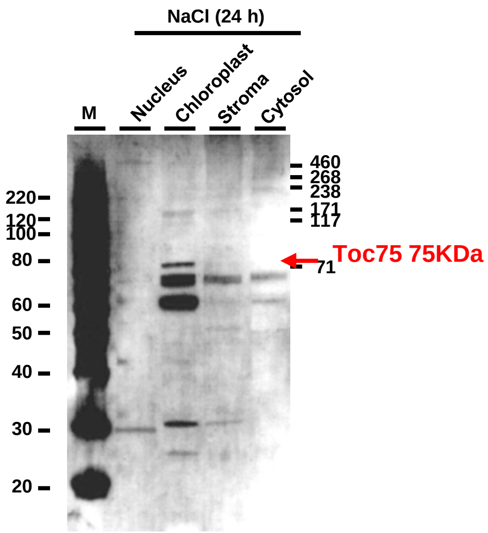

Ab: α-Toc75

Third Row: Western blot with α-RbcL

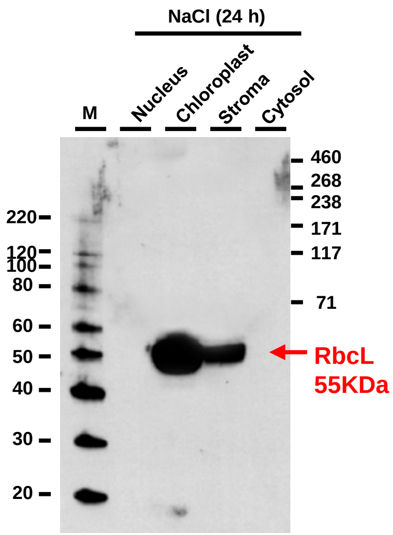

Ab: α-RbcL

Forth Row: Western blot with α-Actin

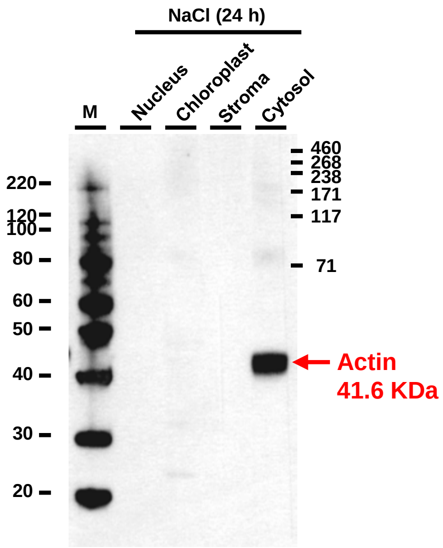

Ab: α-Actin

The full-length blot of Fig. 6b,
Equal loading with α-RBCL

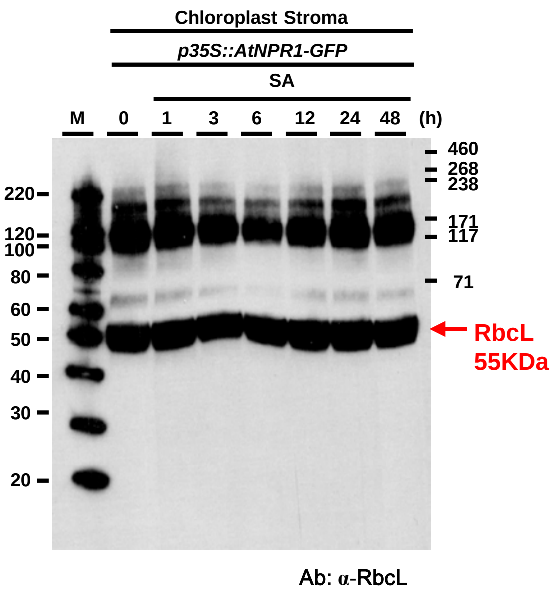

The full-length blot of Fig. 6c,
Equal loading with α-RBCL

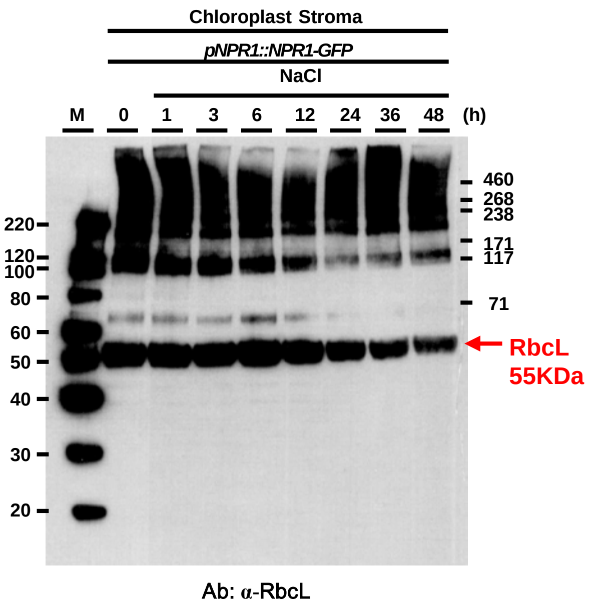

The full-length blot of Fig. 6d,
Equal loading with α-His3

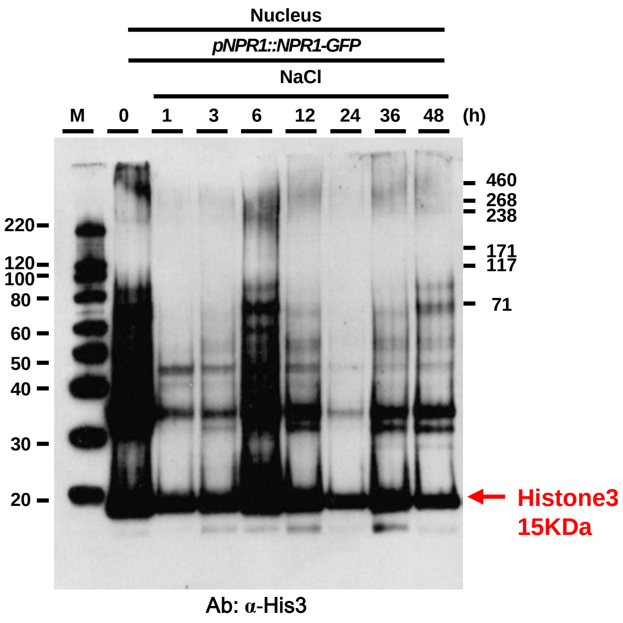

Supplement: Supplementary file 1 — Supplementary information. [file 41598_2020_61379_MOESM1_ESM.pdf]
